# Evolutionary Analysis of Inter-Farm Transmission Dynamics in a Highly Pathogenic Avian Influenza Epidemic

**DOI:** 10.1371/journal.ppat.1002094

**Published:** 2011-06-23

**Authors:** Arnaud Bataille, Frank van der Meer, Arjan Stegeman, Guus Koch

**Affiliations:** 1 Department of Farm Animal Health, Faculty of Veterinary Medicine, Utrecht University, Utrecht, The Netherlands; 2 Department of Virology, Central Veterinary Institute, Animal Sciences Group, Wageningen University and Research Centre, Lelystad, The Netherlands; Erasmus Medical Center, Netherlands

## Abstract

Phylogenetic studies have largely contributed to better understand the emergence, spread and evolution of highly pathogenic avian influenza during epidemics, but sampling of genetic data has never been detailed enough to allow mapping of the spatiotemporal spread of avian influenza viruses during a single epidemic. Here, we present genetic data of H7N7 viruses produced from 72% of the poultry farms infected during the 2003 epidemic in the Netherlands. We use phylogenetic analyses to unravel the pathways of virus transmission between farms and between infected areas. In addition, we investigated the evolutionary processes shaping viral genetic diversity, and assess how they could have affected our phylogenetic analyses. Our results show that the H7N7 virus was characterized by a high level of genetic diversity driven mainly by a high neutral substitution rate, purifying selection and limited positive selection. We also identified potential reassortment in the three genes that we have tested, but they had only a limited effect on the resolution of the inter-farm transmission network. Clonal sequencing analyses performed on six farm samples showed that at least one farm sample presented very complex virus diversity and was probably at the origin of chronological anomalies in the transmission network. However, most virus sequences could be grouped within clearly defined and chronologically sound clusters of infection and some likely transmission events between farms located 0.8–13 Km apart were identified. In addition, three farms were found as most likely source of virus introduction in distantly located new areas. These long distance transmission events were likely facilitated by human-mediated transport, underlining the need for strict enforcement of biosafety measures during outbreaks. This study shows that in-depth genetic analysis of virus outbreaks at multiple scales can provide critical information on virus transmission dynamics and can be used to increase our capacity to efficiently control epidemics.

## Author Summary

Outbreaks of highly pathogenic avian influenza (HPAI) viruses have affected poultry worldwide in the last decades, resulting in vast socioeconomic damages and many human infections. It is important to determine the route of transmission between poultry farms to be able to implement efficient control measures. Here, we investigate possible use of sequence data to unravel the route of virus transmission during an HPAI H7N7 epidemic that took place in 2003 in the Netherlands. We obtained virus sequence data from most of the outbreaks during the epidemic, and found a high level of genetic diversity driven by a rapid evolutionary rate of HPAI H7N7 virus. The phylogenetic inference of the inter-farm transmission network turned out to be difficult due to the presence of potential reassortant virus strains, multiple mutations at highly variable sites and within farm virus diversity. However, most virus samples could be grouped within clearly defined and chronologically sound clusters of infection, giving us valuable insights on the diffusion of the virus during the outbreak. We discuss the implications of the results obtained for the evolutionary and epidemiological dynamics of avian influenza viruses and disease control.

## Introduction

Highly pathogenic avian influenza (HPAI) viruses represent a major concern for public health and global economy, as outbreaks in the last decades resulted in vast socioeconomic damages and numerous human infections. Thanks to increasing availability of avian influenza virus sequence data and the development of new computational and statistical methods of analysis, phylogenetic studies have largely contributed to a better understanding of the emergence, spread and evolution of HPAI epidemics [1]–[3]. However, sampling of genetic data has never been used or dense enough to allow detailed studies of a single outbreak [4]. The rapid evolutionary dynamics of avian influenza viruses suggest that sufficient genetic diversity may be produced during an outbreak in poultry to permit the reconstruction of the inter-flock transmission network, providing important insights for the implementation of efficient control measures. Notably, such detailed genetic data could be used in combination with epidemiological data to study the dynamics of epidemic spread, as has been done for the 2001 food-and-mouth disease outbreak in the UK [5]. However, much remains to be learned about the way evolutionary processes, such as natural selection or reassortment, shape avian influenza virus diversity during an epidemic and how these processes could affect the inference of virus transmission dynamics [4]. We also expect that successful identification of inter-farm transmission pathways depend on the extent and structure of intra-flock and intra-animal viral genetic variation, but perhaps most notably on the size of the virus population bottleneck in the process of inter-farm transmission [4].

The epidemic of HPAI H7N7 in the Netherlands in 2003 represents a unique opportunity to study the epidemiological and evolutionary processes involved in HPAI transmission dynamics in detail. This epidemic started in the most poultry-dense area of the Netherlands (Gelderse valley, Gelderland province) on February 28, 2003. Despite implementation of control measures, the outbreak spread across the entire Gelderland area as well as in a contiguous central region with a lower density of poultry farms. New outbreaks were reported in April in the Limburg province, another poultry-dense area in the South of the Netherlands, in Germany and in Belgium [6]. A total of 255 Dutch farms became infected in a 9 weeks period, and more than 30 million birds were culled during the course of the epidemic [6]. The virus was transmitted to 89 people who were directly involved in handling of infected poultry [7], including one veterinarian who died after developing acute respiratory distress syndrome [8]. Detailed data gathered during the epidemic (e.g. location, date of suspicion and sampling, type of farm, culling date) have been used to estimate epidemiological parameters characterizing this epidemic, notably the spatial range over which the virus spread between farms [9]. However, the transmission route between farms could not be resolved, leaving critical questions about the mechanisms of virus transmission and the efficiency of control measures unanswered.

The H7N7 virus was sampled from the majority of the 255 farms infected, but, to date, only little genetic data have been published from this epidemic [8], [10]. In this study, we present virus sequence data from 72% of the farms infected during the 2003 HPAI H7N7 epidemic in the Netherlands. Phylogenetic analyses were used to unravel the pathways of virus transmission between farms and between outbreak areas. In addition, we investigated the evolutionary processes (substitution rate, selection pressure, reassortment etc.) that were shaping the H7N7 genetic diversity. We also examined the within-flock viral sequence variation on selected farms using clonal sequencing to assess its impact on our phylogenetic analyses. Finally we discuss the implications of the obtained results on our knowledge of the evolutionary and epidemiological dynamics of avian influenza viruses and consequences for disease control.

## Results

### High levels of genetic diversity in HPAI H7N7

**Figure 1:**
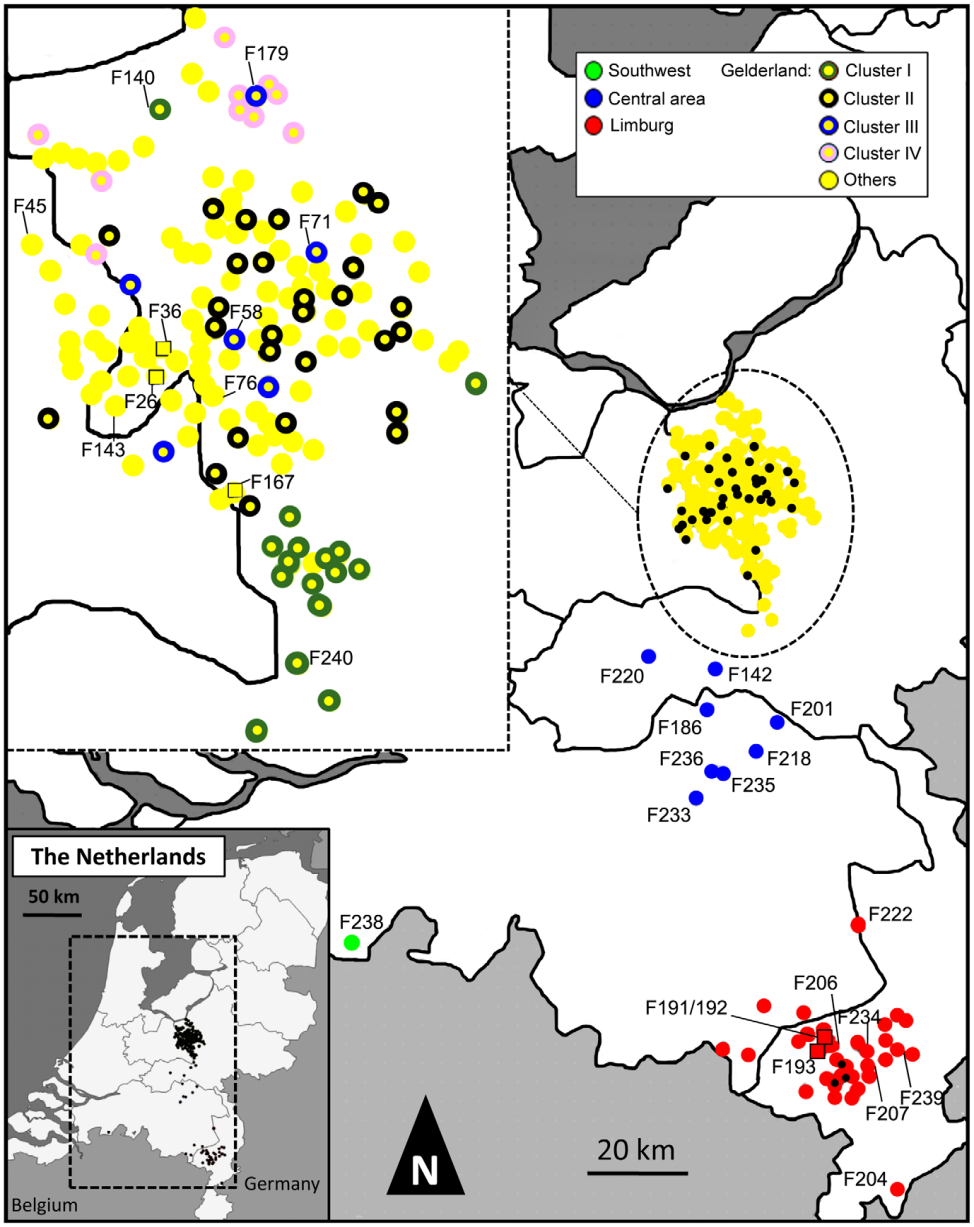
Map indicating the locations of farms infected during the 2003 HPAI H7N7 epidemic. Farms are represented by coloured dots, according to their location and inclusion in a cluster of infection. Black dots in the main map correspond to farm samples not analyzed in this study. Farm samples represented by coloured squares were used for the within-flock viral genetic analyses. In order to maintain the clarity of the figure, only the names of the farms mentioned in the main text are shown. All samples are described in details in Table S1.

Virus RNA was extracted from homogenized trachea tissue samples from dead chickens (5 chickens per sample) obtained from 184 of the 255 farms infected during the H7N7 outbreak (72% coverage of the epidemic, Figure 1). We could not process more samples due to logistical constraints, but we considered that this coverage was sufficient to reach the aims of this study. The viral sequence datasets consist of full-length sequences of the H7-hemagglutinin (HA), N7-neuraminidase (NA) and basic polymerase 2 (PB2) gene segments; preliminary analysis of five full viral genomes previously obtained from humans and chickens infected at early and late stages of the H7N7 outbreak (available in public databases) showed that these three genes contain the highest level of genetic diversity among the 8 gene segments (data not shown). Farms are labelled from F1 to F255, following the order of sample submission to the laboratory during the outbreak. Samples were selected for sequencing in order to cover the entire timeline and all areas of the epidemic (Gelderland, Limburg, central area and southwest area; Figure 1). Moreover, all farms infected within 7 days before the first report of infection in the Limburg area (April 3, 2003) were analysed in an attempt to find the source of this new outbreak. Details of location and date of sample collection, and GISAID accession numbers are listed for each sample in Table S1. The HA, NA and PB2 sequences of the human fatal case (A/Netherlands/219/03, [8]) were included in the final dataset.

A total of 74 substitution sites were recovered in HA, defining 71 sequences among which 50 were unique in the dataset. NA was less polymorphic (59 substitution sites), but a strand of 52 to 74 nucleotides in the NA stalk region was also found deleted in 13 samples from the Limburg area, with a total of 7 different types of deletions, 3 of which resulted in a frame shift in the NA coding sequence (Table S1). In total, the complete NA sequence dataset defined 64 different genotypes (42 singletons). The PB2 sequence data had the highest number of polymorphic sites (81), defining 64 different genotypes (38 singletons). The combination of the genetic data from the three genes permitted us to define farm specific genotypes for 141 out of the 184 farms (76%). The HA, NA and PB2 sequence datasets were found to be free of homologous recombination using Recombination Detection Program version 2 (RDP2) [11].

### Rapid evolutionary rate and early origin of HPAI H7N7

Rates of nucleotide substitution and time of most recent common ancestor (TMRCA) of the HPAI H7N7 viruses were estimated separately for the three gene datasets using a Bayesian Markov Chain Monte Carlo (BMCMC) method [12] as implemented in BEAST [13], using sampling dates to calibrate the molecular clock (Table S1). Bayes Factors (BF) [14] were used to select among strict and relaxed clock models of evolution [15], and among demographic models of population growth. The relaxed uncorrelated exponential clock model associated with an exponential growth model fitted better the data (Table S2). The analyses showed that the mean substitution rate was very high for both HA and NA datasets (1.18×10^−2^ and 1.02×10^−2^ substitutions per site per year (substitutions/site/year), respectively; Table 1), whereas the estimated rate for the PB2 dataset was twice lower (0.54×10^−2^ substitutions/site/year). These estimates were associated with large 95% highest posterior density intervals (HPD; Table 1). TMRCA estimations showed that the origin of the HPAI H7N7 virus dated back to mid-January 2003 according to the HA dataset, and as far back as late December and late October 2002 for the NA and PB2 datasets, respectively (Table 1). Again, estimations from the NA and PB2 datasets were affected by large HPD intervals. Similar estimations of substitution rates and TMRCA were obtained with other sub-optimal clock and demographic models (Table S2), showing that these results are robust and not artefacts of the priors used in the Bayesian analyses.

**Table 1:** Mean nucleotide substitution rates and estimation of TMRCA of the H7N7 epidemic.

	BMCMC analysis			
Gene	Mean substitution rate (×10^−2^)	Substitution rate HPD (×10^−2^)	Mean TMRCA	HPD TMRCA
HA	1.18	0.79–1.59	15/01/2003	05/12/2002–06/02/2003
NA	1.02	0.65–1.42	25/12/2002	24/10/2002–09/02/2003
PB2	0.54	0.34–0.74	20/10/2002	14/03/2002–13/01/2003

HPD, 95% highest posterior density intervals. Dates are presented in day/month/year.

### Phylogenetic analyses

**Figure 2:**
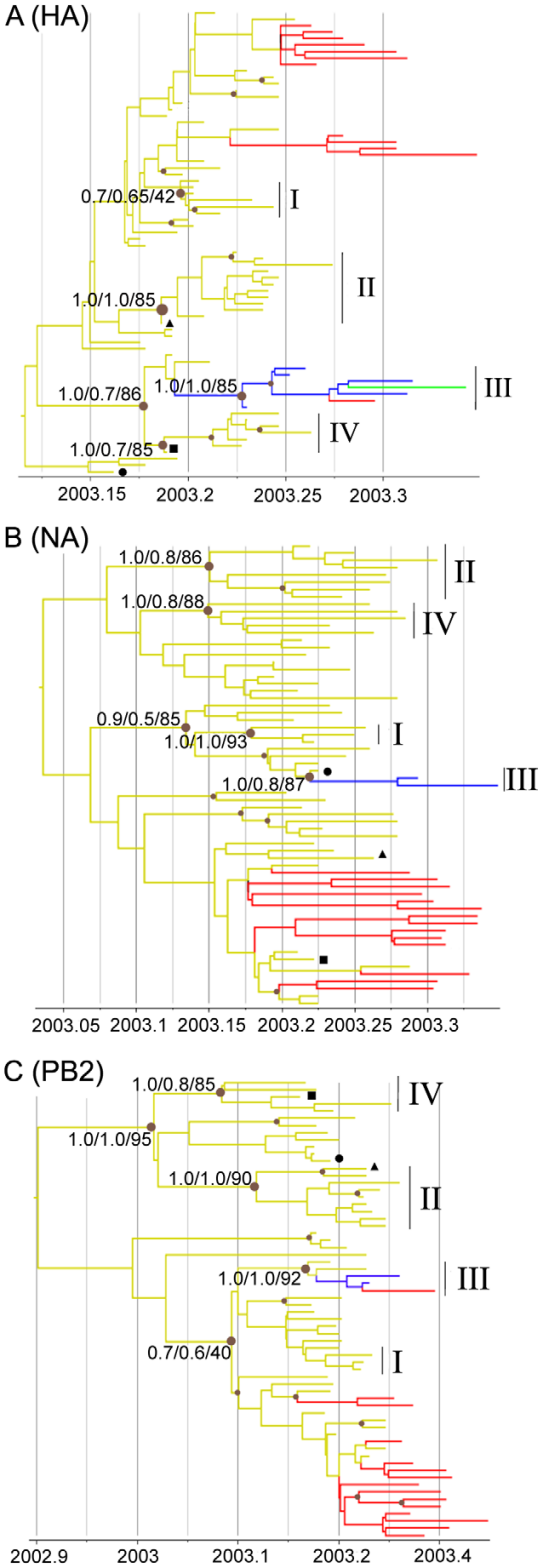
Phylogenetic trees of H7N7 viruses. Time-scaled phylogenies (dates on the horizontal axis) inferred using Bayesian MCMC analysis from (A) HA gene; (B) NA gene; (C) PB2 gene. Nodes supported by ≥0.7 posterior probability are indicated by a grey dot. Posterior probability values from the time-scaled BMCMC method, the MrBayes BMCMC method, and the Maximum Likelihood method (1,000 ML bootstrap replications) are shown for nodes delimitating clusters of transmission (tsBMCMC/MrBMCMC/ML; noted Cluster I–IV). The three samples with discordant phylogenies are indicated by black square (F45), circle (F76), and triangle (F145). Nodes and branches are coloured according the geographical origin of the farm samples. Yellow, Gelderland area; Blue, Central area; Red, Limburg area; Green, Southwest area. Fully annotated trees are available online in supplementary figures S2A–C.

Phylogenetic trees of the HPAI H7N7 virus sequences were reconstructed for the three separate HA, NA and PB2 sequence datasets using Bayesian Inference and Maximum Likelihood methods (Figure 2A–C, Figure S1A–C). For each gene phylogeny, multiple sequences could be grouped in clusters that were well supported statistically. Notably, we could identify 4 clusters of sequences present in all gene phylogenies (Cluster I–IV, Figure 2A–C). Three of these clusters regrouped virus samples from farms infected in the Gelderland area only (Cluster I, II, IV; in yellow in Figure 2A–C, Table S1), whereas Cluster III included all samples from the outbreaks in the Central area (blue labelling), the fatal human case, a sample from the outbreak in the southwest of the Netherlands (green labelling; F238; Figure 1), the sample from the most northern outbreak in the Limburg area (red labelling, F222), and some samples from outbreaks in the Gelderland area (Figure 2A–C, Table S1).

**Figure 3:**
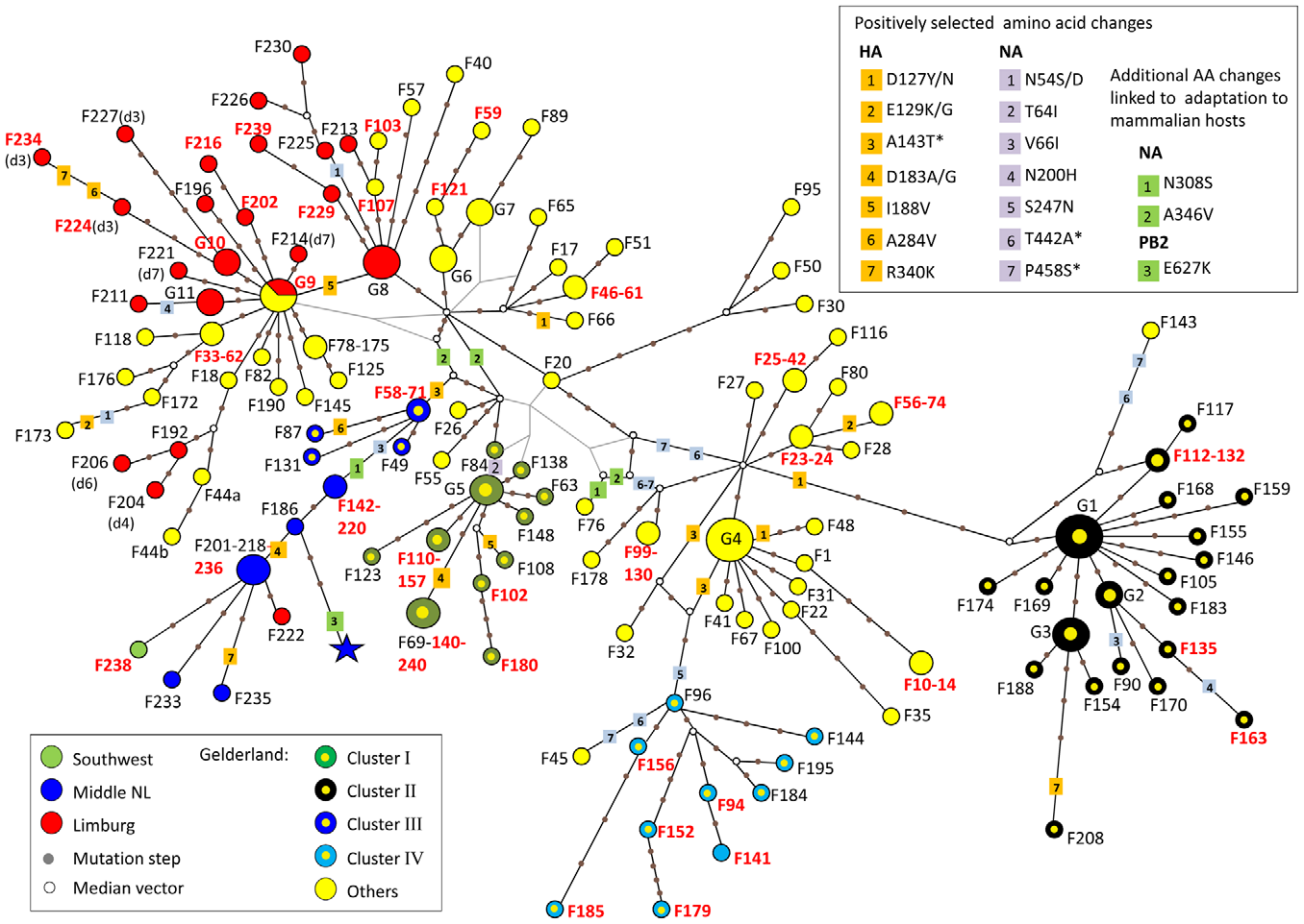
Median-joining phylogenetic network of H7N7 viruses. The median-joining network was constructed from the combined HA, NA and PB2 sequence data. This network includes all the most parsimonious trees linking the sequences. Each unique sequence genotype is represented by a coloured circle sized relative to its frequency in the dataset. Genotypes are coloured according to the location of the farm sample and its inclusion in a cluster of infection. Branches in black represent the shortest trees; Additional branching pathways are in grey. Each node is separated by a specific number of mutations represented by grey dots. Mutations corresponding to specific amino acid changes are indicated. For genotypes containing a deletion in the NA stalk region, the type of deletion is indicated between brackets beside the name of the isolate (see Table S1 for the description of deletion types). Names of farm samples involved in likely inter-farm transmission events are in red (see Table 2). (*) positively selected amino acids linked to adaptation to mammalian hosts. **G1**: group of samples including F38, F54, F64, F113, F162, F194, F199; **G2**: F134, F160, F166; **G3**: F122, F161, F164, F171, F182; **G4:** F2, F5, F12, F21, F43, F60, F91; **G5:** F39, F70, F92, F129; **G6:** F15, F29, F37; **G7:** F16, F19, F52; **G8**: F193, F217, F223, F231; **G9**: F36, F68, F167, F191(d1), F205(d5), F207(d2); **G10**: F203 (d3), F219(d3), F228(d3); **G11**: F197, F242, F232.

To assess the inter-farm transmission network, we manually concatenated the HA, NA and PB2 sequences for all virus samples, and used this single alignment to construct a Median Joining phylogenetic network [16] with the program NETWORK [17] (Figure 3). The network obtained included all the most parsimonious trees, thus represented all the plausible evolutionary pathways linking the farm samples. The network showed that most virus sequences were grouped in multiple clusters of infection, including the 4 transmission clusters identified with the gene-specific phylogenetic analyses (Figure 3). Sequences within these 4 clusters were separated in average by 3–4 nucleotide differences, whereas 11–20 differences were observed between clusters. All clusters were connected at the base of the network by complex reticulations that rendered the relationships between the clusters hard to determine. In most cases, one virus sequence identified in multiple farms was at the origin of an infection cluster. All Limburg samples (apart from F222) were grouped with Gelderland samples in 2 clusters that were separated by one single mutation step. These 2 clusters presented some chronological anomalies (Figure S2). Notably, the network showed that a virus strain from a farm (F18) that had been culled a month before the first infection in Limburg was the closest ancestor to a group of 3 Limburg samples (F192, F204 and F206; Figure 3, Table S1, Figure S2). Also, according to the network, 4 virus strains that emerged in Gelderland during the first 3 weeks of the epidemic (F40, F57, F103 and F107) would have originated from a virus strain that infected farms in Limburg after the 5^th^ week of the epidemic.

We identified 15 pairs of farm samples that uniquely shared identical sequence genotypes, representing likely transmission events (Table 2, Figure 3). Furthermore, we could identify 13 pairs of samples that were unambiguously connected in the phylogenetic network. Of these 28 likely inter-farm transmission events, 25 involved farms located in the same infected area, with a distance of 0.8–13.6 Km separating them (Table 2). The three remaining transmission events linked farms separated by much larger distances (31.3–84.4 Km). The two longest transmission events corresponded to a transmission from a farm in Gelderland to the index farm of Limburg (F167–F191, distance: 84.4 Km), and from a farm in the central area to a farm located is the southwest of the Netherlands (F236–F238, distance: 65.9 Km; Table 2, Figure 1).

**Table 2:** Summary of the most likely transmission events identified either from pair of farm samples exclusively sharing the same sequence genotype, or pair of farm samples having sequence genotypes unambiguously linked in the network analysis.

Identical genotypes			Direct network connections		
Sample pair	Location	Distance (km)	Sample pair	Location	Distance (km)
F10-F14	G-G	1.1	F59-F121	G-G	13.6
F23-F24	G-G	7.4	F94-F141	G-G	2.9
F25-F42	G-G	8.2	F102-F180	G-G	13.3
F33-F62	G-G	2.1	F103-F107	G-G	11.2
F46-F61	G-G	2	F135-F163	G-G	2.6
F56-F74	G-G	12.4	F152-F179	G-G	1.4
F58-F71	G-G	4.2	F156-F185	G-G	3.3
F99-F130	G-G	1.2	F172-F173	G-G	2.6
F110-F157	G-G	1.9	F202-F216	L-L	2
F111-F132	G-G	3.1	F207-F219	L-L	10.2
F142-F220	C-C	12.3	F224-F234	L-L	3.4
F219-F228	L-L	1.1	F229-F239	L-L	0.8
F36-F68	G-G	5.7	**F236-F238**	**C-S**	**65.9**
**F140-F240**	**G-G**	**31.3**			
**F167-F191**	**G-L**	**84.4**			

Probable long distance transmission events are in bold. C, Central area; G, Gelderland; L, Limburg; S, Southwest area.

### Selection pressure and molecular characterization in the HA, NA and PB2 genes

We assessed the selection pressures acting on the three genes by estimating the ratio of non-synonymous to synonymous nucleotide substitutions (*ω*  = *d*_N_ */d*_S_) in the different datasets using in CODEML [18]. When averaged over all sites, all three genes were predominantly affected by neutral or purifying selection (*ω* <1), with PB2 under the strongest negative natural selection (*ω* = 0.313; Table 3). Additionally, likelihood ratio tests revealed that a model allowing site-specific positive selection pressure (M2a in CODEML) fitted significantly better than a model of nearly neutral selection (M1a) for the HA gene (*p* = 0.031; Table 3). A Bayes Empirical Bayes analysis [19] identified 7 amino acid sites in HA that were under positive selection (residues 127, 129, 143, 183, 188, 284, 340), although none of these sites were supported by a significant posterior probability value (*Pr*<0.95). We further tried to identify sites under positive selection in the three genes using the single-likelihood ancestor counting (SLAC), the random effect likelihood (REL), and the fixed effect likelihood (FEL) methods [20]. The SLAC and FEL methods failed to detect positive selection in any of the three genes. The REL method identified the same 7 amino acid sites in HA already detected with CODEML as under positive selection with high posterior probability values (*Pr*>0.95). In addition, it detected 7 amino acid sites under positive selection in the NA dataset (residues 54, 64, 66, 200, 247, 442, 458).

**Table 3:** Values of Log-likelihood (lnL) and *d*_N_ */d*_S_ for HA, NA and PB2 genes using different selection models in the CODEML analysis, and LRT tests comparing the two models.

	M1a (nearly neutral)		M2a (positive selection)		LRT (M2a-M1a)	
Gene	lnL	*d*_N_ */d*_S_	lnL	*d*_N_ */d*_S_	2Δl	*p*-values
HA	−3031.91	0.545	−3028.44	0.736	6.94	*0.031*
NA	−2449.32	0.493	−2448.46	0.578	1.72	0.423
PB2	−3788.85	0.313	−3788.85	0.313	0	1.000

We used the degree of freedom of 2 for these LRT tests that is expected to be too conservative.

The biological functions of most of these positively selected residues in the HA and NA molecules are not known. Only the A143T substitution, which introduces a new potential N-linked glycosylation site in HA, has previously been identified as being associated with enhanced virulence in avian hosts [21]. Also, three positively selected amino acid changes, A143T in HA, and T442A and P458S in NA, have been also detected in the human fatal case [8], and have been shown to contribute in enhanced replication efficiency of the HPAI H7N7 virus in mammalian hosts [10]. The A143T substitution was found in virus samples from 27 different farms (Table S1; Figure 3). The T442A and P458S amino acid changes in NA were present in the majority of the farm samples (113 farms). Three other amino acid changes identified in the human fatal case and linked to enhanced replication in mammalian hosts (E627K in PB2; N308S and A346V in NA [10]) were not found to be positively selected. The N308S and A346V in NA were identified in 12 and 36 farm samples, respectively, as well as in the fatal human case (Table S1; Figure 3). The E627K in PB2 was not found in chicken samples.

### Identification of potential reassortant viruses

**Figure 4:**
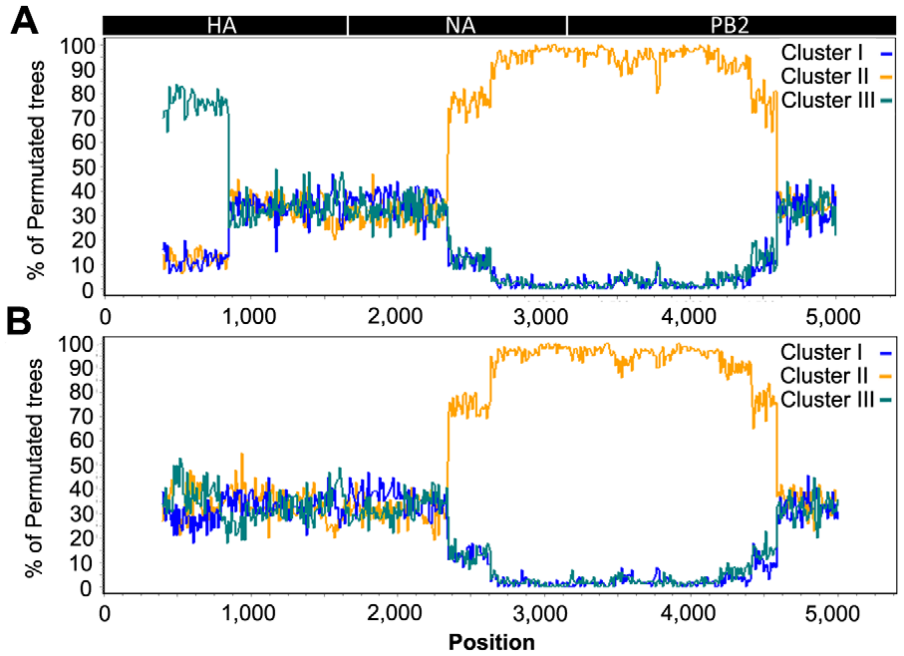
Recombination analysis on concatenated H7N7 virus sequences. (A) Bootscan analysis on the full dataset; (B) Bootscan analysis on the dataset with the HA codon 143 removed. The Cluster IV virus group was used as query in the analysis, with an 800 bp window size and step size of 10 bp. A schematic diagram of the concatenated HA, NA and PB2 virus segments is shown on top.

We found discrepancies in the phylogenetic relationship between the four identified transmission clusters in the HA, NA and PB2 phylogenies. Cluster III was closely related to Cluster IV in the HA phylogeny, but Cluster III was closely related to Cluster I in the NA and PB2 phylogenies, and Cluster IV closely related to Cluster II in the PB2 phylogeny (Figure 2A–C). These discordances suggest that one or more of the transmission clusters originated from reassortment events. We further investigated the putative reassortant viruses using bootscan analyses [22] on a selected dataset (n = 50) of manually concatenated HA-NA-PB2 sequences (Figure 4A–B, Figure S3A–D; see methods). Results of the bootscan plot showed that Cluster IV was highly similar to Cluster III in the HA segment, but clustered with Cluster II in the NA and PB2 segments (Figure 4A). The graph did not produce a clear-cut breakpoint between the HA and the NA-PB2 segments, probably because of the poor level of genetic diversity in some gene regions. We noticed that sequences grouped in the Cluster III and IV were all characterized by the presence of the A143T amino acid change in their HA gene (Figure 3, Table S1). Removing the codon position 143 from the HA dataset resulted in the loss of support for the clustering of these two groups of sequences in the phylogenetic trees and the bootscan analysis, thus for the signal of reassortment (Figure 4B).

In addition, we also observed that the placement of the sequence of three Gelderland farm samples differed between the NA phylogeny and the HA and PB2 phylogenies (F45, F76 and F143; Figure 2A–C). None of the bootscan analyses performed on these three samples showed a significant signal for recombination (Figure S3A–C). Similarly to the potential reassortant event detected for Cluster III and IV, the F45, F76 and F143 sequences were characterized by the presence of positively selected amino acid changes in NA (Table S1, Figure 3).

### Within flock viral genetic diversity

**Figure 5:**
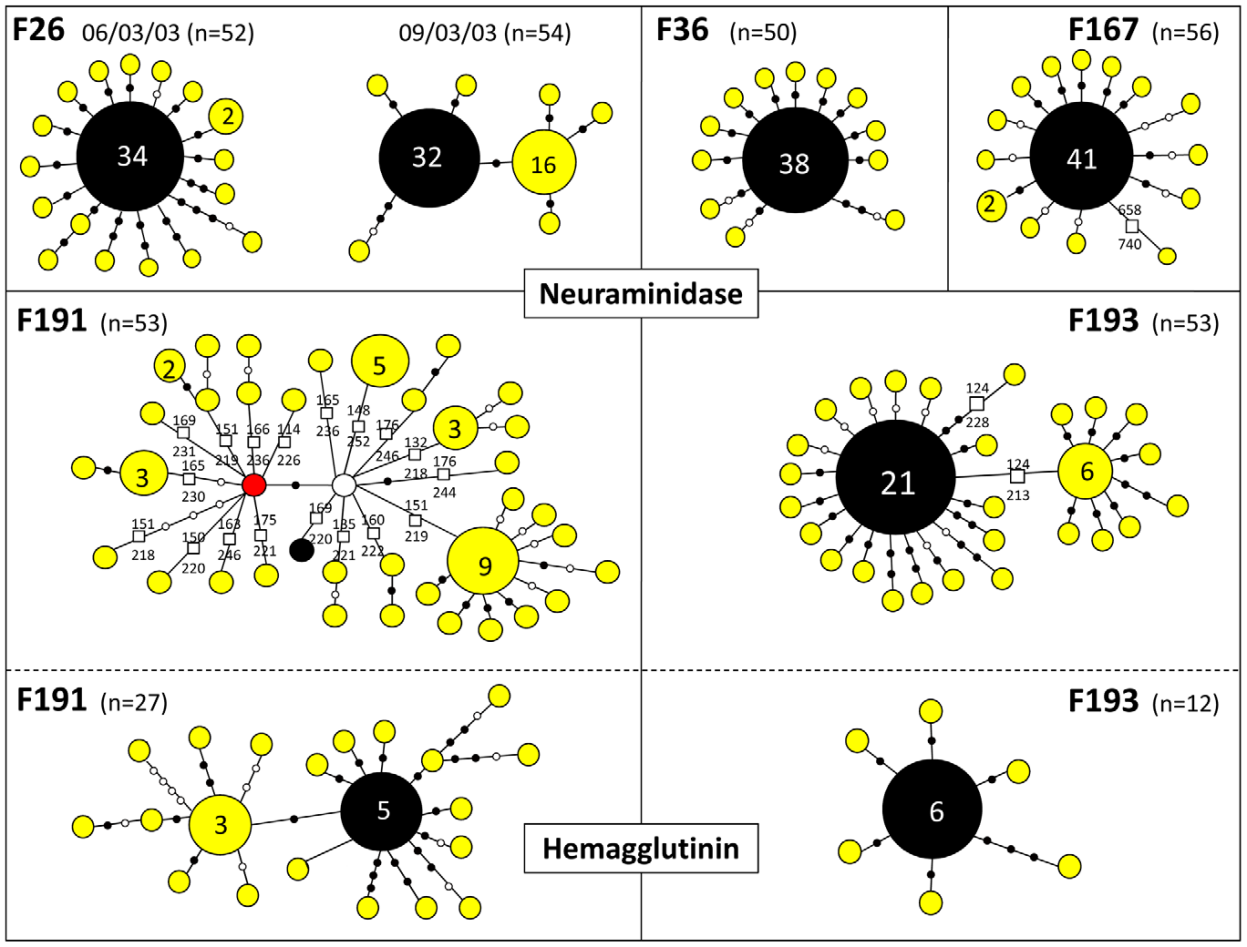
Schematic diagrams summarizing within-flock genetic diversity in 6 farm samples. The sequence variants found by clonal sequencing of partial NA and HA genes in 6 different samples are represented by coloured circles sized relatively to their frequency. Total number of clones sequenced per sample (n) is indicated. The exact number of copies of each genetic variant is indicated when >1. Variants in black correspond to the sequence originally isolated in each farm. Each variant is separated by nucleotide substitutions represented by filled black dots (non-synonymous changes) or open dots (synonymous changes), and by deletions represented by squares. The exact position of the deletion in the NA gene is indicated. The red node represents a variant similar to the sequence obtained for the F192 sample (see main text). The white node represents a potential missing variant.

To estimate the viral genetic diversity within hosts and within flocks, we performed clonal sequencing targeting an 850 bp portion of the NA gene (position 57–908) on 6 farm samples (5 chickens per sample). We chose 4 samples (F36, F167, F191 and F193; Figure 1) positioned at the base of the Limburg-Gelderland transmission clusters in the network (within groups G8 and G9 in Figure 4) in order to further assess the origin of the Limburg outbreak and of the chronological anomalies detected in the network. We also performed clonal sequencing on the F26 farm (Figure 1), because two samples taken three days apart (March 6, and March 9, 2003) were available for this farm, allowing us to assess changes in viral genetic diversity within a flock. A total of 50–54 clones with NA inserts were sequenced per sample (Table 4, Figure 5). We performed an additional clonal sequencing analysis targeting a 570 bp portion of the HA gene (bases 81–650), but, due to poor cloning success, only 27 clones with HA inserts could be obtained from F191 and 12 clones from F193.

**Table 4:** Summary of results obtained from clonal sequencing.

Sample	N	H	% Dom	d_S_	d_N_	deletion
**NA**						
F26 (March 6)	52	18	65.4	2	21	0
F26 (March 9)	54	8	59.3	1	7	0
F36	50	13	76	7	10	
F167	56	15	73.2	8	9	1(1)
F191	53	35	17	14	11	52 (18)
F193	53	28	39.6	8	25	8(2)
**HA**						
F191	27	21	18.5	12	26	0
F193	12	7	50	0	7	0

N, number of clones sequenced; H, total number of sequence variants identified; % Dom, percentage of the clones with the dominant sequence variant; d_S_, number of synonymous substitutions; d_N_, number of non-synonymous substitutions; deletion, number of variants found with a deletion (number of different type of deletions).

For all the 4 samples tested, the most abundant NA and HA sequence variant represented 17–76% of the obtained sequences (Table 4, Figure 5). With the exception of the NA variants obtained for F191, this dominant variant was identical to the sequence originally obtained and used in the general HA and NA datasets. The other identified sequence variants were usually present at low frequency (most of the time only once) and directly linked to the dominant variant, differing from it by 1–4 nucleotide substitutions (Figure 5). The clonal diversity of NA in F26 (March 9, 2003) and F193, and of HA in F191 were characterised by the presence of another sequence variant at relatively high frequency and at the origin of low-frequency variants. The diversity of NA sequence variants was extremely high in F191, with all variants but one containing a stalk deletion (18 different types of deletion were identified; Table 4; Figure 5).

A total of 168 nucleotide substitutions were recorded, among which 116 were non-synonymous substitutions (Table 4, Figure 5). Only 5 out of 168 substitutions had already been identified in the HA and NA epidemic datasets. Notably, 14 of the 35 NA variants identified in F191 shared a mutation that had been only found in farm samples F44, F192, F204 and F206, establishing a link that was missing between the index farm of Limburg and the three Limburg farms (F192, F204, F206; red node Figure 5; Figure 3).

## Discussion

This study presents one of the most complete viral genetic data ever obtained on a highly pathogenic avian influenza epidemic, with coverage of 72% of the poultry farms that were infected during the 2003 HPAI H7N7 epidemic in the Netherlands. Results obtained in this study showed that the HA, NA and PB2 gene segments were characterized by a high level of genetic diversity, allowing the identification of unique virus sequences for 76% of the farm samples analyzed. The estimates of substitution rates for the HA and NA gene averaged around 1×10^−2^ substitutions per site per year, which is among the highest observed for avian influenza viruses [23]. It suggests that enough viral genetic diversity can be produced within a short period of time during an HPAI epidemic to allow the use of partial genome sequences to determine virus transmission dynamics with phylogenetic analyses.

Analyses of selection pressure showed that this rapid evolutionary rate was mainly driven by a combination of neutral evolution and purifying selection pressure, with only a limited amount of site-specific positive selection pressure identified in the HA and NA genes. TMRCA estimations indicate that the H7N7 virus may have been introduced in poultry weeks before the first mortality was reported. This is in agreement with epidemiological models based on mortality data indicating that approximately two weeks can elapse after introduction of H7N7 in a flock before change in mortality is observed [24]. The presence of identical virus sequences in multiple farms infected at different time periods (e.g. 6 farms in group G9 spanning a period of more than 5 weeks) also suggests that the HPAI H7N7 virus was already very stable and well adapted to poultry when the epidemic started. In addition, the phylogenetic network showed that many amino acid changes associated with increased pathogenicity in mammals appeared already at an early stage during the epidemic. These results have serious implications for disease control, as they demonstrate that early and regular monitoring of poultry farms is necessary to detect and contain avian influenza viruses before they fully adapt to domestic poultry and become a potential risk for animal and public health.

We identified several other evolutionary processes that could have affected the observed viral genetic diversity and might have led to misleading results in our phylogenetic analyses. Firstly, the presence of reassortant viruses in our dataset could provoke poor resolution or even false identification of farm-to-farm transmission events. Three virus strains (F45, F76 and F143) and one cluster of closely related viruses (Cluster IV) were identified as potential reassortants due to their discordant position in the phylogenetic trees and the network. However, the signal of reassortment in all sequences was closely associated with signal of positive selection at specific amino acid residues, so the discordances in the phylogenies may be due to convergent evolution driven by the adaptive advantages conferred by these amino acid changes. In all cases, we prefer to consider these farm samples unsuitable for the study of the H7N7 inter-farm transmission dynamics until more is known about these possible reassortment events.

Secondly, an important limitation of our genetic dataset is the characterisation of only one virus sequence per farm, whereas each farm (and each individual host within this farm) may contain a wide variety of closely related virus variants. Our genetic data could be considered a reliable tool to elucidate the transmission pathways of the HPAI H7N7 between farms if it can be assumed that the virus genotype obtained for each farm samples represents the dominant strain in the farm sample and that this dominant strain is the one most likely to be transmitted to other farms. These assumptions were partially supported by clonal sequencing performed on six farm samples, as the genotype used in our dataset corresponded to the dominant variant in the clone population in 5 out of 6 farms. This dominant variant represented >50% of the clones in 4 samples (Table 4), suggesting that more cloning effort would most probably not change this result. The identification of a second sub-dominant strain directly related to the dominant strain in two samples suggests that dominance can evolve during the course of infection within a flock. This evolution of dominance could have caused the genetic differences observed between farm samples directly connected in the network. These results have, however, to be considered with caution because of the small number of farm tested with the cloning technique and of the very small sampling relative to flock size obtained from each farm (5 chickens per farm).

Importantly, clonal sequencing of the one farm sample (F191) also showed that the virus strain originally sequenced was not the dominant variant of the farm but was a potentially inactive variant (as it contained a frame shift deletion) present at a low frequency. It suggests that our genetic dataset was not always composed of the dominant genotype in the farm samples, potentially affecting the resolution of the transmission network. The variant with highest frequency in sample F191 represented 17% of the clones, suggesting that the absence of a highly dominant strain may have allowed the sequencing of another variant. This lack of dominance could be due to the production of a high variety of variants with stalk deletion, possibly associated with the evolution of a deletion-prone polymerase in viruses infecting the first farm in the Limburg area (F191). If it is the case, it would suggest that only the 13 samples presenting deletions in our dataset may have been wrongly positioned in the phylogenetic network due to the dominance issue.

Most mutations differentiating the multiple genetic variants from the dominant variant in the clone populations were associated with amino acid changes or deletions, suggesting that the virus population within a flock (and possibly within a single individual) is composed of strains of variable fitness, with one or few best-fit strains dominating the population. We cannot rule out the possibility that the genetic variation observed in our cloning results is an artefact of RNA manipulation. However, the error rate of the RT polymerase used (SuperScript III, Invitrogen, Carlsbad CA, USA) was estimated to be 1/15,000 by the manufacturer, so it should not have a major influence on an analysis targeting an 800 bp gene region. Also, the genetic variation we obtained is similar to what has been observed in other avian Influenza viruses using a similar approach [25], or in Hepatitis C Virus using a pyrosequencing approach [26].That 97% of the mutations identified in all clone variants examined were not found in the complete epidemic dataset suggests that inter-farm transmission of H7N7 was accompanied by a population bottleneck. It is important to note that this analysis was realized with a small number of farm samples, and that the small number of chicken sampled per farm greatly limited our capacity to assess properly the viral diversity within flocks. A larger study, probably involving a pyrosequencing approach [27] and experimental infections in controlled environment, would be necessary to further tackle the issues of intra- and inter- host viral genetic diversity and transmission bottlenecks in HPAI.

Results from the network and the clonal sequencing analysis of F191 showed that some mutations occurred multiple times at different time periods, leading to chronological anomalies in the farm-to-farm connections identified in the phylogenetic network. These anomalies were limited to clusters including Limburg samples, suggesting that the high viral genetic diversity produced during the outbreak in Limburg may be at their origin. Interestingly, reports from officials involved in the control of the epidemic indicate that F191 may have been infected for over a week before being reported and sampled. Also this farm housed >10,000 turkeys, a species shown to play a key role in the evolution of AI pathogenicity in domestic animals [28], [29]. Only few other turkey farms were infected during the epidemic, and their culling was swift according to the reports. Therefore, it is likely that the long infection period of the F191 turkey farm is at the origin of its high genetic diversity and of many anomalies in the phylogenetic network. Additional cloning work may help resolving all chronological anomalies in the network. An interesting alternative would be to combine our genetic network with temporal data (and other epidemiological data) in a mathematical framework to calculate the likelihood of potential transmission events, as it has been done for the 2001 food-and-mouth disease outbreak in the UK [5].

Overall, our results suggest that a combination of evolutionary processes, such as multiple mutations at highly variable sites, positive selection, and/or reassortment, drove the genetic diversity observed in the HPAI H7N7. The effect of these processes might have been stronger at the early stages of the epidemic, as farms may have been infected for longer time before control measures were taken (as reported for F191). The long branches and poor quality of connections at the base of the network supports this hypothesis. Despite this complex evolutionary history of the H7N7 virus, most farm samples could be grouped within clearly defined and chronologically sound clusters of infection, giving us valuable insights on the spreading of the virus during the epidemic.

### Inter-farm transmission dynamics of HPAI H7N7

Boender *et al.* [9] have previously performed a spatial analysis of inter-farm transmission using epidemiological data from the HPAI H7N7 epidemic. They showed that risk of transmission decreased with inter-farm distance and they could map higher-risk areas for the spread of the virus. However, the epidemiological data did not permit the resolution of the pathways of transmission between farms. Our results show that the analysis of viral genetic data can complement epidemiological studies, allowing notably the identification of clusters of infections and of specific farm-to-farm transmission events. The geographical position of the farms associated with the transmission clusters identified from the phylogenetic analyses is indicated in Figure 1. Most of the farms of Cluster I are located geographically close to one another, suggesting that inter-farm virus transmission during the epidemic was at least partially caused by short distance air-borne movements of virus particles [30]. However, farms of cluster III showed a combination of aggregated and dispersed geographical location, whereas Cluster II and Cluster III were more dispersed within the Gelderland area. It is possible that such transmission between farms separated by 5–15 km occurred naturally, as avian influenza viruses could persist for long periods in the environment (e.g. water or feather) [31], [32]. The poultry production system with its professional contacts could also have favoured virus spread during the epidemic. Notably, some operations of farms, such as egg collection, were resumed during the epidemic and may have played a role in virus transmission [33]. We estimated that the H7N7 virus was present in poultry maybe weeks before the first outbreak, so it is possible that the virus spread via a network of contacts formed by normal poultry operations across the Gelderland area before the implementation of the transport ban. Also, it has been recently shown that many humans involved in the control of the epidemic were infected by H7N7 in farms they visited [34]. Thus, H7N7 virus might have been transmitted between farms by infected people or by human-mediated mechanical transport. Analyzing the sequence of virus isolated from infected humans and the movements of people involved in control activities could help to determine whether human-mediated transport played a role in the inter-farm transmissions.

Importantly, our results also suggest that a discrete number of long distance transmission events were at the origin of the virus spread into new areas, rather than a slow wave-like movement of the virus towards the south of the country. Notably, it is interesting to note that farm UN167, a back-yard poultry farm, seems to be at the origin of the outbreak in the Limburg area. Conversely, Bavink *et al.* [35] showed with epidemiological models that back-yard poultry probably played a marginal role during the outbreak, suggesting that pre-emptive culling of this type of farm may not always be necessary. Our results suggest that these types of poultry farms should still be considered important in control strategies. This result and all other farm-to-farm transmission events identified should be considered with caution because 27% of the farms infected during the epidemic could not be sequenced. However, only few missing farms could still be at the origin of the infection in Limburg and all are located in Gelderland >50 km away from the index farm of Limburg (Figure 1). It strongly suggests that a long distance transmission event is at the origin of the second important H7N7 outbreak in the Netherlands. Such long distance movements of avian influenza are most likely the results of human-mediated transport of the virus, although airborne spread cannot be totally ruled out. Possible causes of human-mediated virus movements are lack of knowledge or poor compliance of the biosafety measures implemented, such as unauthorized movements of birds or their products. Better enforcement and more widely distributed biosafety instructions and training could substantially decrease the risk of introduction of the virus to new areas. Future studies combining genetic data with available epidemiological data should provide a better resolution of the inter-farm transmission network that shaped the epidemic, and further understanding on the mechanisms involved in H7N7 spread during the epidemic. This study shows that partial viral genomic data (here 3 genes out of the 8 composing the AI genome) can provide important insights on the transmission dynamics of HPAI viruses even at the scale of temporally and spatially limited epidemic. Our study also strongly suggests that comprehensive study of the evolutionary processes involved in shaping virus diversity are needed in order to use viral genetic data in such ways.

## Materials and Methods

### Viral sequence data

Samples were collected as part of the diagnosis by veterinarians of the Food and Consumer Product Safety Authority (the Netherlands) and submitted to the Central Veterinary Institute for confirmation by virus isolation. The authors of this study were not involved in sample collection. Viral RNA was directly extracted from 184 infected trachea tissue samples from dead chickens (5 chickens per sample) using a High Pure Viral RNA extraction kit (Roche Diagnostics Indianapolis IN, USA). Complementary DNA was synthesized by reverse transcription reaction using SuperScript III (Invitrogen, Carlsbad CA, USA), and gene amplification by PCR was performed using the PCR Expand high fidelity kit (Roche Diagnostics Indianapolis IN, USA) and primers specific for the hemagglutinin (HA), neuraminidase (NA) and basic polymerase 2 (PB2) gene segments. Sequencing was performed by using the BigDye Terminator v. 1.1. sequencing kit (Applied Biosystems, Foster City CA , USA) and an ABI Prism 3130 genetic analyzer (Applied Biosystems). Primers and PCR protocols are detailed in Table S4. Nucleotide sequences are available from the GISAID database (EPI_ISL_68268-68352, and EPI_ISL_82373-82472; Table S1).

### Phylogenetic analyses

Sequences were aligned using BIOEDIT [36]. We used likelihood ratio tests, Akaike and Bayesian information criteria as implemented in DataMonkey [37] to select the simplest evolutionary model that best fit the different dataset. For the three genes, a Hasegawa–Kishino–Yano (HKY) model with gamma-distributed rates among sites was selected. Rates of nucleotide substitution and time of most recent common ancestors (TMRCAs) were estimated for the three genes using a Bayesian Markov Chain Monte Carlo (BMCMC) method [12] as implemented in the program BEAST [13]. Isolation dates were used to calibrate the molecular clock. Different combinations of molecular clock (strict clock or uncorrelated relaxed clocks [15]) and demographic models were attempted independently and the best-fit clock and demographic models were selected by performing Bayes factor tests [14]. The limited timespan of our samples required the use of a simple model to avoid over-parameterization [38], so we used a single HKY model over all sites in preference to a codon-partitioned model for these analyses. For each dataset, three independent runs were conducted for 60 million generations, sampling every 2,000 generations. Convergences and effective sample sizes of the estimates were checked using TRACER [39]. Trees were summarized in a maximum clade credibility (MCC) tree after a 20% burn-in using TREEANNOTATOR [13]. The resulting time-scaled phylogenetic trees were visualised with FIGTREE [40].

Additional methods were used to infer the phylogenetic relationships from the HA, NA and PB2 datasets. A Bayesian MCMC inference method was performed in MRBAYES [41], with multiple runs of 10 million generations with a 20% burn-in, sampling every 100 generations, and using the default heating parameters. A Maximum Likelihood (ML) method implemented in PHYML [42] was also used with a bootstrap analysis of 1,000 full bootstrap replicates to test the robustness of tree topologies. The three gene segment alignments were manually concatenated to generate a single alignment that was used to construct a phylogenetic network using the Median Joining method [16] implemented in the program NETWORK [17]. This model-free method uses a parsimony approach, based on pairwise differences, to connect each sequence to its closest neighbour, and allows the creation of internal nodes (“median vectors”), which could be interpreted as unsampled or extinct ancestral genotypes to link the existing genotypes in the most parsimonious way. The parameter *epsilon*, which controls the level of homoplasy, was set at the same value as the weight of characters used to calculate the genetic distances (weight value  = 10). The average number of nucleotide differences within and between the phylogenetic clusters identified was calculated with MEGA [43].

### Recombination and reassortment detection

Homologous recombination within each gene segment was searched using Recombination Detection Program version 2 (RDP2) [11]. Putative reassortant viruses were preliminarily identified by the topological incongruity between transmission clusters identified across the phylogenies of different gene segments (see results). This was further investigated with a subset of virus sequences including samples from transmission cluster I (n = 9), cluster II (n = 19), cluster III (n = 8), and cluster IV (n = 10), from Limburg area (n = 10), and four additional samples with incongruent phylogenies (F45, F76, F143, F210). For each sample, the sequences from the 3 genes were manually concatenated, and the resulting alignment was analyzed using bootscan analyses [22] implemented in SIMPLOT [44].

### Detection of selection pressure

Selection pressure on the HA, NA and PB2 genes was investigated by estimating the ratio of non-synonymous to synonymous nucleotide substitutions (*ω* = *d*_N_ */d*_S_) using codon-based phylogenetic methods implemented in CODEML (available in the PAML package [18]). Likelihood ratio tests (LRTs) were used to test whether model M1a of neutral evolution (sites restricted to 0<*ω*<1) or model M2a of positive selection (allows sites with *ω* >1) was a statistically better fit to the data [45]. If the null model M1a was rejected in preference of M2a, a Bayes Empirical Bayes method was used to identify individual codons under positive selection [19]. In addition, positively selected sites were detected using the single-likelihood ancestor counting (SLAC), the random effect likelihood (REL), and the fixed effect likelihood (FEL) methods [20] via the Datamonkey website [37].

### Within flock viral genetic diversity

PCR amplification targeting a 850 bp portion of the NA gene (bases 57–908) was performed on cDNA obtained from five samples (F26, F36, F167, F191 and F193) using the PCR Expand high fidelity kit (Roche Diagnostics). An additional PCR was used to amplify a 570 bp portion of the HA gene (bases 81–650) on the two samples from Limburg only (F191, F193). Primers and PCR protocols are described in Table S4. PCR products were purified using the High Pure PCR Products Purification kit (Roche Diagnostics), and were cloned using the pGEM-T Easy Vector System (Promega, Madison WI, USA). Clones with inserts of the correct size were identified by agarose gel electrophoresis. A total of 50–56 clones with NA inserts were sequenced per farm sample using the BigDye Terminator sequencing kit, version 1.1. and an ABI Prism 3130 genetic analyzer (Applied Biosystems). Nucleotide sequences are available from the GISAID database (EPI_ISL_82561-82902). Sequences were aligned to the original HA or NA sequence obtained for each farm samples using BIOEDIT [36], and nucleotide differences were recorded manually.

## Supporting Information

Figure S1 Time-scaled phylogenies (dates on the horizontal axis) inferred using Bayesian MCMC analysis from (A) HA gene; (B) NA gene; (C) PB2 gene. Nodes supported by >0.7 posterior probability are indicated by a grey dot. Posterior probability values from the time-scaled BMCMC method, the MrBayes BMCMC method, the Maximum Likelihood method (1,000 ML bootstrap replications) are also indicated (tsBMCMC/MrBMCMC/ML). Samples sharing identical HA, NA, and/or PB2 (named as “Group1-11” in the trees, when >3 isolates) are listed in Table S3.
(TIF)

Figure S2 Time-scaled median-joining phylogenetic network of H7N7 viruses. This network is identical to figure 3, but with each farm sample positioned along a time axis starting a day zero of the epidemic. See legend of Figure 3 for more details.
(TIF)

Figure S3 Bootscan recombination analysis on the full dataset of concatenated H7N7 virus sequences. Sequences obtained from (A) F45, (B) F76, and (C) F143 were used as query in the analysis, with a 800 bp window size and step size of 10 bp. A schematic diagram of the concatenated HA, NA and PB2 virus segments is shown on top.
(TIF)

Table S1 Description of the H7N7 virus samples used in this study.
(DOC)

Table S2 Summary statistics of the BMCMC analyses.
(DOC)

Table S3 List of farm isolates sharing identical HA, NA and/or PB2 sequences. Group names refer to names given in phylogenetic trees in Figure S2A–C.
(DOC)

Table S4 List of primers and protocols used for PCR amplification and sequencing of HA, NA and PB2 genes.
(DOC)
