# Supplementary figures and images for: Evolutionary Analysis of Inter-Farm Transmission Dynamics in a Highly Pathogenic Avian Influenza Epidemic
Source: PLoS Pathog. 2011 Jun 23;7(6):e1002094. doi: 10.1371/journal.ppat.1002094 (PMC3121798; doi:10.1371/journal.ppat.1002094)

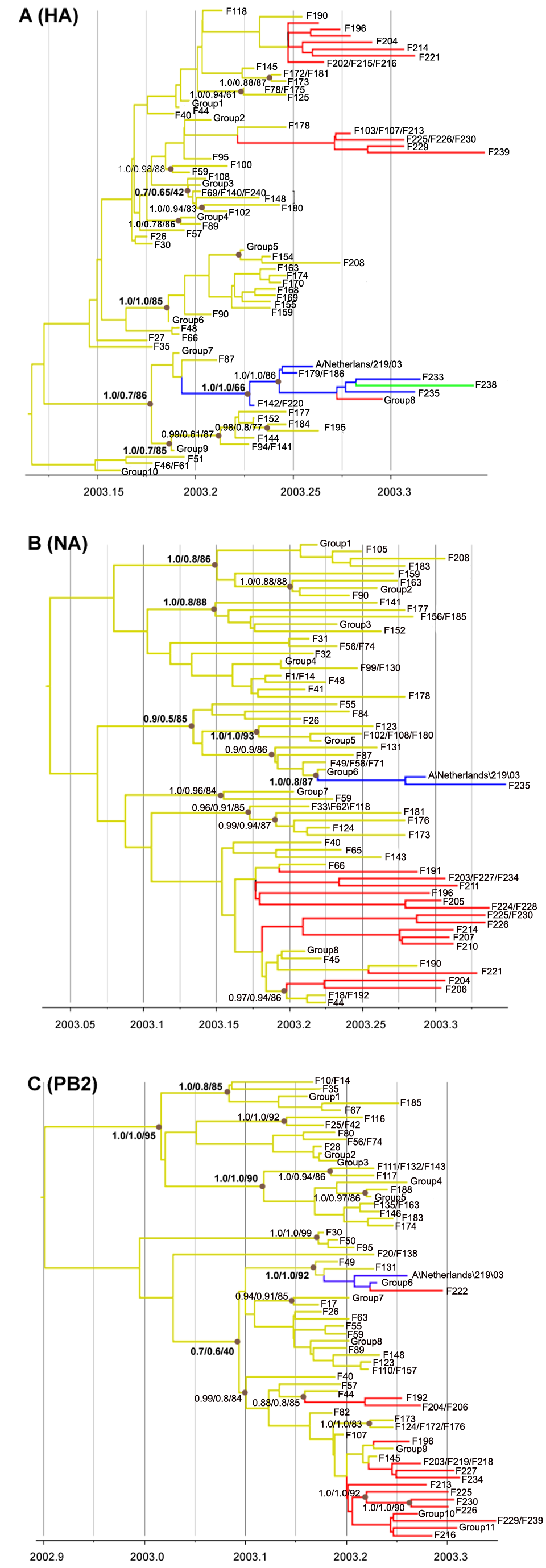

Supplement: Figure S1 — Time-scaled phylogenies (dates on the horizontal axis) inferred using Bayesian MCMC analysis from (A) HA gene; (B) NA gene; (C) PB2 gene. Nodes supported by >0.7 posterior probability are indicated by a grey dot. Posterior probability values from the time-scaled BMCMC method, the MrBayes BMCMC method, the Maximum Likelihood method (1,000 ML bootstrap replications) are also indicated (tsBMCMC/MrBMCMC/ML). Samples sharing identical HA, NA, and/or PB2 (named as “Group1-11” in the trees, when >3 isolates) are listed in Table S3. (TIF) [file ppat.1002094.s001.tif]

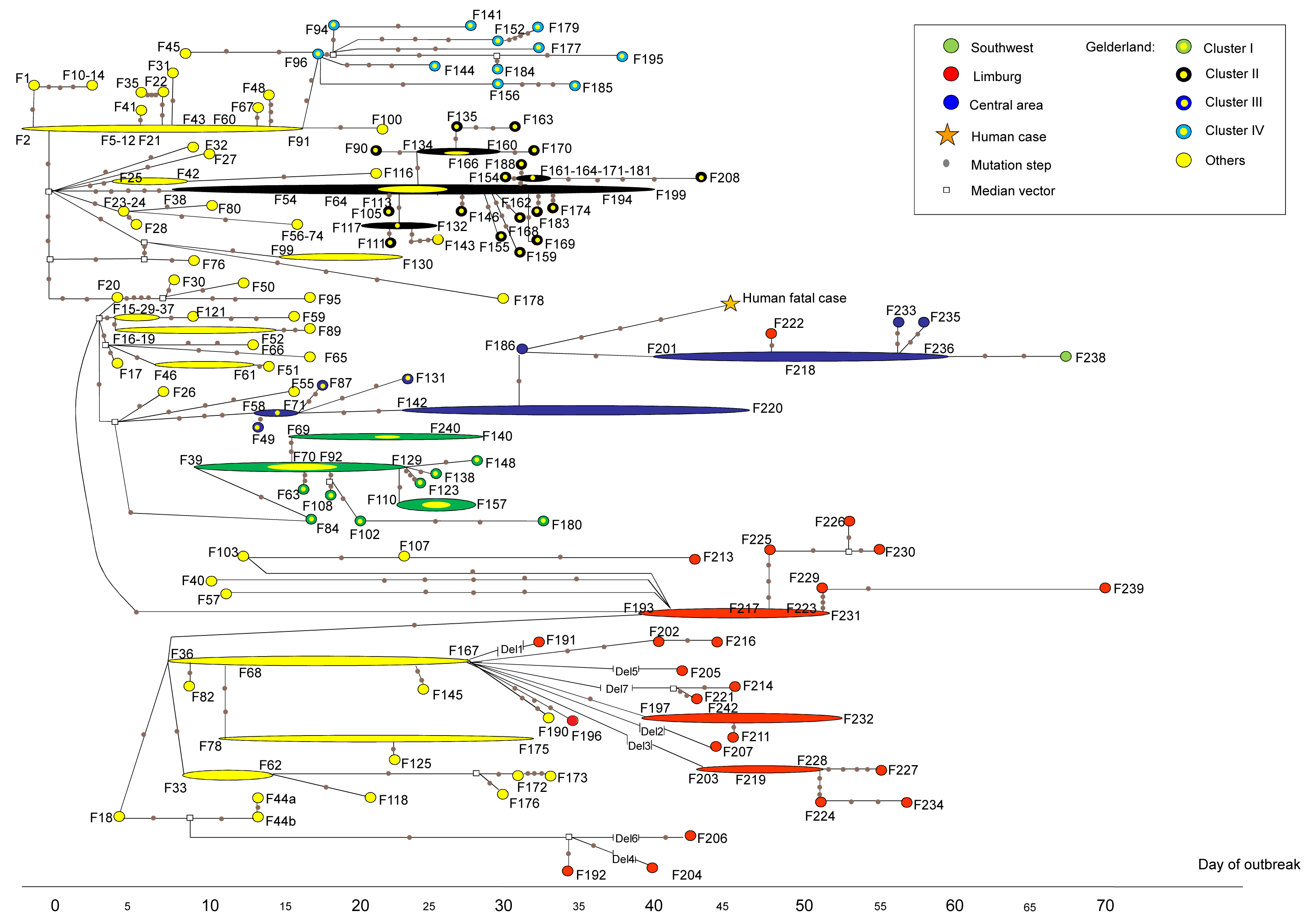

Supplement: Figure S2 — Time-scaled median-joining phylogenetic network of H7N7 viruses. This network is identical to figure 3, but with each farm sample positioned along a time axis starting a day zero of the epidemic. See legend of Figure 3 for more details. (TIF) [file ppat.1002094.s002.tif]

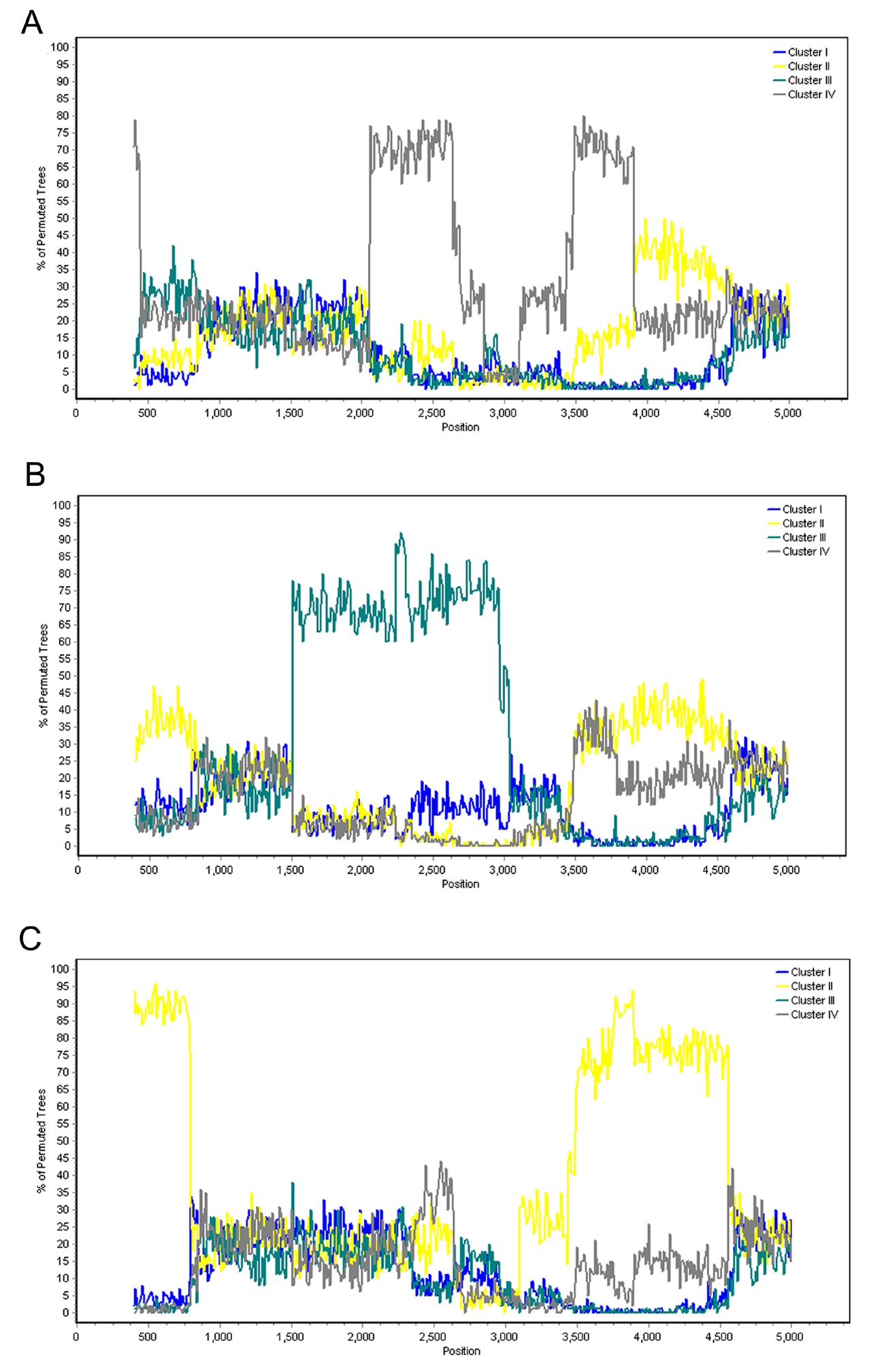

Supplement: Figure S3 — Bootscan recombination analysis on the full dataset of concatenated H7N7 virus sequences. Sequences obtained from (A) F45, (B) F76, and (C) F143 were used as query in the analysis, with a 800 bp window size and step size of 10 bp. A schematic diagram of the concatenated HA, NA and PB2 virus segments is shown on top. (TIF) [file ppat.1002094.s003.tif]
