# Supplementary material for: Evolutionary Analysis of Inter-Farm Transmission Dynamics in a Highly Pathogenic Avian Influenza Epidemic
Source: PLoS Pathog. 2011 Jun 23;7(6):e1002094. doi: 10.1371/journal.ppat.1002094 (PMC3121798; doi:10.1371/journal.ppat.1002094)
Supplement: Table S1 — Description of the H7N7 virus samples used in this study. (DOC) [file ppat.1002094.s004.doc]

**Table S1** Description of the H7N7 virus isolates used in this study.

| Farm code | Sampling date | culling date | Location | Deletion | Cluster | HA A143T | NA N308S | NA A346V | NA T442A | NA P458S | GISAID accession |
| --- | --- | --- | --- | --- | --- | --- | --- | --- | --- | --- | --- |
| 1 | 28/02 | 05/03 | G |  |  |  |  |  |  |  | EPI_ISL_82373 |
| 2 | 28/02 | 06/03 | G |  |  |  |  |  |  |  | EPI_ISL_82374 |
| 5 | 28/02 | 05/03 | G |  |  |  |  |  |  |  | EPI_ISL_67934 |
| 8* | 01/03 | 05/03 | G |  |  |  |  |  | x | x | EPI_ISL_82375 |
| 10 | 02/03 | 05/03 | G |  |  |  |  |  |  |  | EPI_ISL_82376 |
| 12 | 02/03 | 07/03 | G |  |  |  |  |  |  |  | EPI_ISL_68268 |
| 14^+^ | 02/03 | 07/03 | G |  |  |  |  |  |  |  | EPI_ISL_68269 |
| 15 | 03/03 | 05/03 | G |  |  |  |  |  | x | x | EPI_ISL_82377 |
| 16 | 04/03 | 07/03 | G |  |  |  |  |  | x | x | EPI_ISL_82378 |
| 17 | 04/03 | 06/03 | G |  |  |  |  |  | x | x | EPI_ISL_82379 |
| 18 | 04/03 | 07/03 | G |  |  |  |  |  | x | x | EPI_ISL_82380 |
| 19 | 04/03 | 07/03 | G |  |  |  |  |  | x | x | EPI_ISL_82381 |
| 20 | 05/03 | 07/03 | G |  |  |  |  |  | x | x | EPI_ISL_82382 |
| 21 | 05/03 | 07/03 | G |  |  |  |  |  |  |  | EPI_ISL_82383 |
| 22 | 05/03 | 08/03 | G |  |  |  |  |  |  |  | EPI_ISL_82384 |
| 23 | 05/03 | 07/03 | G |  |  |  |  |  |  |  | EPI_ISL_82385 |
| 24 | 05/03 | 07/03 | G |  |  |  |  |  |  |  | EPI_ISL_82386 |
| 25 | 06/03 | 08/03 | G |  |  |  |  |  |  |  | EPI_ISL_68270 |
| 26 | 05/03 | 12/03 | G |  |  |  |  | x | x | x | EPI_ISL_68279 |
| 27 | 05/03 | 10/03 | G |  |  |  |  |  |  |  | EPI_ISL_82387 |
| 28 | 05/03 | 08/03 | G |  |  |  |  |  |  |  | EPI_ISL_82388 |
| 29 | 05/03 | 08/03 | G |  |  |  |  |  | x | x | EPI_ISL_82389 |
| 30 | 06/03 | 08/03 | G |  |  |  |  |  | x | x | EPI_ISL_68280 |
| 31^+^ | 07/03 | 15/03 | G |  |  |  |  |  |  |  | EPI_ISL_82390 |
| 32 | 08/03 | 11/03 | G |  |  | x |  |  |  |  | EPI_ISL_82391 |
| 33 | 07/03 | 10/03 | G |  |  |  |  |  | x | x | EPI_ISL_82392 |
| 35 | 06/03 | 08/03 | G |  |  |  |  |  |  |  | EPI_ISL_68281 |
| 36 | 06/03 | 10/03 | G |  |  |  |  |  | x | x | EPI_ISL_82393 |
| 37^+^ | 06/03 | 07/03 | G |  |  |  |  |  | x | x | EPI_ISL_82394 |
| 38 | 09/03 | 10/03 | G |  | II |  |  |  |  |  | EPI_ISL_68289 |
| 39 | 10/03 | 12/03 | G |  | I |  |  | x | x | x | EPI_ISL_68300 |
| 40* | 10/03 | 12/03 | G |  |  |  |  |  | x | x | EPI_ISL_68282 |
| 41 | 06/03 | 08/03 | G |  |  |  |  |  |  |  | EPI_ISL_82395 |
| 42 | 08/03 | 11/03 | G |  |  |  |  |  |  |  | EPI_ISL_82396 |
| 43* | 09/03 | 12/03 | G |  |  |  |  |  |  |  | EPI_ISL_82397 |
| 44 | 11/03 | 13/03 | G |  |  |  |  |  | x | x | EPI_ISL_82398 |
| 45 | 10/03 | 12/03 | G |  |  | x |  |  | x | x | EPI_ISL_68306 |
| 46 | 06/03 | 08/03 | G |  |  |  |  |  | x | x | EPI_ISL_82399 |
| 48 | 11/03 | 14/03 | G |  |  |  |  |  |  |  | EPI_ISL_68308 |
| **Table S1** Description of the H7N7 virus isolates used in this study (continued). | | | | | | | | | | | |
| Farm code | Sampling date | culling date | Location | Deletion | Cluster | HA A143T | NA N308S | NA A346V | NA T442A | NA P458S | GISAID accession |
| 49 | 11/03 | 13/03 | G |  | III | x |  | x | x | x | EPI_ISL_68310 |
| 50 | 11/03 | 13/03 | G |  |  |  |  |  | x | x | EPI_ISL_82400 |
| 51 | 12/03 | 14/03 | G |  |  |  |  |  | x | x | EPI_ISL_82401 |
| 52 | 12/03 | 15/03 | G |  |  |  |  |  | x | x | EPI_ISL_82402 |
| 54 | 13/03 | 15/03 | G |  | II |  |  |  |  |  | EPI_ISL_82403 |
| 55 | 14/03 | 16/03 | G |  |  |  |  | x | x | x | EPI_ISL_68271 |
| 56 | 14/03 | 16/03 | G |  |  |  |  |  |  |  | EPI_ISL_68272 |
| 57 | 12/03 | 14/03 | G |  |  |  |  |  | x | x | EPI_ISL_68283 |
| 58* | 11/03 | 12/03 | G |  | III | x |  | x | x | x | EPI_ISL_82404 |
| 59 | 13/03 | 16/03 | G |  |  |  |  |  | x | x | EPI_ISL_82405 |
| 60 | 01/03 | 11/03 | G |  |  |  |  |  |  |  | EPI_ISL_82406 |
| 61 | 11/03 | 13/03 | G |  |  |  |  |  | x | x | EPI_ISL_82407 |
| 62 | 15/03 | 16/03 | G |  |  |  |  |  | x | x | EPI_ISL_82408 |
| 63 | 15/03 | 16/03 | G |  | I |  |  | x | x | x | EPI_ISL_68301 |
| 64 | 15/03 | 18/03 | G |  | II |  |  |  |  |  | EPI_ISL_82409 |
| 65 | 15/03 | 17/03 | G |  |  |  |  |  | x | x | EPI_ISL_68284 |
| 66 | 11/03 | 13/03 | G |  |  |  |  |  | x | x | EPI_ISL_68309 |
| 67 | 12/03 | 13/03 | G |  |  |  |  |  |  |  | EPI_ISL_68273 |
| 68 | 13/03 | 16/03 | G |  |  |  |  |  | x | x | EPI_ISL_68313 |
| 69 | 15/03 | 17/03 | G |  | I |  |  | x | x | x | EPI_ISL_68330 |
| 70 | 15/03 | 17/03 | G |  | I |  |  | x | x | x | EPI_ISL_68302 |
| 71* | 15/03 | 19/03 | G |  | III | x |  | x | x | x | EPI_ISL_68311 |
| 74* | 17/03 | 18/03 | G |  |  |  |  |  |  |  | EPI_ISL_68285 |
| 76 | 11/03 | 14/03 | G |  |  |  | x | x | x | x | EPI_ISL_68274 |
| 78 | 04/03 | 11/03 | G |  |  |  |  |  | x | x | EPI_ISL_82410 |
| 80 | 13/03 | 13/03 | G |  |  |  |  |  |  |  | EPI_ISL_82411 |
| 82 | 09/03 | 10/03 | G |  |  |  |  |  | x | x | EPI_ISL_82412 |
| 84 | 17/03 | 20/03 | G |  | I |  |  | x | x | x | EPI_ISL_68303 |
| 87 | 18/03 | 20/03 | G |  | III | x |  | x | x | x | EPI_ISL_82413 |
| 89 | 14/03 | 17/03 | G |  |  |  |  |  | x | x | EPI_ISL_82414 |
| 90 | 17/03 | 22/03 | G |  | II |  |  |  |  |  | EPI_ISL_68299 |
| 91 | 18/03 | 19/03 | G |  |  |  |  |  |  |  | EPI_ISL_82415 |
| 92 | 16/03 | 18/03 | G |  | I |  |  | x | x | x | EPI_ISL_82416 |
| 94 | 14/03 | 22/03 | G |  | IV | x |  |  |  |  | EPI_ISL_68332 |
| 95 | 17/03 | 17/03 | G |  |  |  |  |  | x | x | EPI_ISL_82417 |
| 96 | 17/03 | 17/03 | G |  | IV | x |  |  |  |  | EPI_ISL_82418 |
| 99 | 19/03 | 21/03 | G |  |  |  |  |  |  |  | EPI_ISL_82419 |
| 100^+^ | 20/03 | 23/03 | G |  |  |  |  |  |  |  | EPI_ISL_82420 |
| 102 | 20/03 | 22/03 | G |  | I |  |  | x | x | x | EPI_ISL_68305 |
| **Table S1** Description of the H7N7 virus isolates used in this study (continued). | | | | | | | | | | | |
| Farm code | Sampling date | culling date | Location | Deletion | Cluster | HA A143T | NA N308S | NA A346V | NA T442A | NA P458S | GISAID accession |
| 103* | 15/03 | 15/03 | G |  |  |  |  |  | x | x | EPI_ISL_82421 |
| 105 | 20/03 | 22/03 | G |  | II |  |  |  |  |  | EPI_ISL_68290 |
| 107 | 20/03 | 23/03 | G |  |  |  |  |  | x | x | EPI_ISL_82422 |
| 108 | 16/03 | 20/03 | G |  | I |  |  | x | x | x | EPI_ISL_82423 |
| 110 | 23/03 | 23/03 | G |  | I |  |  | x | x | x | EPI_ISL_82424 |
| 111 | 22/03 | 23/03 | G |  | II |  |  |  |  |  | EPI_ISL_82425 |
| 113 | 22/03 | 23/03 | G |  | II |  |  |  |  |  | EPI_ISL_82426 |
| 116 | 20/03 | 22/03 | G |  |  |  |  |  |  |  | EPI_ISL_82427 |
| 117 | 13/03 | 23/03 | G |  | II |  |  |  |  |  | EPI_ISL_68291 |
| 118 | 19/03 | 23/03 | G |  |  |  |  |  | x | x | EPI_ISL_82428 |
| 121 | 08/03 | 11/03 | G |  |  |  |  |  | x | x | EPI_ISL_82428 |
| 122 | 23/03 | 25/03 | G |  | II |  |  |  |  |  | EPI_ISL_68336 |
| 123 | 23/03 | 24/03 | G |  | I |  |  | x | x | x | EPI_ISL_82429 |
| 125 | 23/03 | 25/03 | G |  |  |  |  |  | x | x | EPI_ISL_68337 |
| 129 | 23/03 | 26/03 | G |  | I |  |  | x | x | x | EPI_ISL_82430 |
| 130 | 23/03 | 25/03 | G |  |  |  |  |  |  |  | EPI_ISL_82431 |
| 131 | 24/03 | 27/03 | G |  | III | x |  | x | x | x | EPI_ISL_68312 |
| 132 | 24/03 | 25/03 | G |  | II |  |  |  |  |  | EPI_ISL_68292 |
| 134 | 24/03 | 25/03 | G |  | II |  |  |  |  |  | EPI_ISL_68293 |
| 135 | 24/03 | 27/03 | G |  | II |  |  |  |  |  | EPI_ISL_82432 |
| 138 | 24/03 | 27/03 | G |  | I |  |  | x | x | x | EPI_ISL_68304 |
| 140* | 24/03 | 30/03 | G |  | I |  |  | x | x | x | EPI_ISL_68331 |
| 141 | 24/03 | 24/03 | G |  | IV | x |  |  |  |  | EPI_ISL_68333 |
| 142 | 25/03 | 26/03 | C |  | III | x | x | x | x | x | EPI_ISL_68339 |
| 143 | 25/03 | 27/03 | G |  |  |  |  |  | x | x | EPI_ISL_68294 |
| 144 | 25/03 | 25/03 | G |  | IV | x |  |  |  |  | EPI_ISL_68334 |
| 145 | 25/03 | 26/03 | G |  |  |  |  |  | x | x | EPI_ISL_68323 |
| 146 | 26/03 | 27/03 | G |  | II |  |  |  |  |  | EPI_ISL_82433 |
| 148 | 26/03 | 28/03 | G |  | I |  |  | x | x | x | EPI_ISL_68352 |
| 152* | 25/03 | 30/03 | G |  | IV | x |  |  |  |  | EPI_ISL_68335 |
| 154 | 28/03 | 30/03 | G |  | II |  |  |  |  |  | EPI_ISL_82434 |
| 155 | 28/03 | 30/03 | G |  | II |  |  |  |  |  | EPI_ISL_82435 |
| 156 | 28/03 | 30/03 | G |  | IV | x |  |  |  |  | EPI_ISL_68307 |
| 157 | 28/03 | 28/03 | G |  | I |  |  | x | x | x | EPI_ISL_82436 |
| 159 | 28/03 | 31/03 | G |  | II |  |  |  |  |  | EPI_ISL_82437 |
| 160 | 28/03 | 30/03 | G |  | II |  |  |  |  |  | EPI_ISL_82438 |
| 161 | 28/03 | 31/03 | G |  | II |  |  |  |  |  | EPI_ISL_82439 |
| 162 | 29/03 | 31/03 | G |  | II |  |  |  |  |  | EPI_ISL_82440 |
| 163 | 29/03 | 31/03 | G |  | II |  |  |  |  |  | EPI_ISL_82441 |
| 164 | 29/03 | 31/03 | G |  | II |  |  |  |  |  | EPI_ISL_82442 |
| **Table S1** Description of the H7N7 virus isolates used in this study (continued). | | | | | | | | | | | |
| Farm code | Sampling date | culling date | Location | Deletion | Cluster | HA A143T | NA N308S | NA A346V | NA T442A | NA P458S | GISAID accession |
| 166 | 29/03 | 01/04 | G |  | II |  |  |  |  |  | EPI_ISL_68295 |
| 167^+^ | 27/03 | 28/03 | G |  |  |  |  |  | x | x | EPI_ISL_82443 |
| 168 | 29/03 | 31/03 | G |  | II |  |  |  |  |  | EPI_ISL_82444 |
| 169 | 29/03 | 01/04 | G |  | II |  |  |  |  |  | EPI_ISL_82445 |
| 170 | 30/03 | 01/04 | G |  | II |  |  |  |  |  | EPI_ISL_82446 |
| 171 | 30/03 | 31/03 | G |  | II |  |  |  |  |  | EPI_ISL_82447 |
| 172 | 30/03 | 31/03 | G |  |  |  |  |  | x | x | EPI_ISL_82448 |
| 173 | 31/03 | 02/04 | G |  |  |  |  |  | x | x | EPI_ISL_82449 |
| 174 | 31/03 | 02/04 | G |  | II |  |  |  |  |  | EPI_ISL_82450 |
| 175 | 31/03 | 01/04 | G |  |  |  |  |  | x | x | EPI_ISL_68338 |
| 176 | 31/03 | 31/03 | G |  |  |  |  |  | x | x | EPI_ISL_68314 |
| 177 | 31/03 | 01/04 | G |  |  |  |  |  | x | x | EPI_ISL_68340 |
| 178 | 31/03 | 01/04 | G |  |  |  |  |  |  |  | EPI_ISL_82451 |
| 179 | 31/03 | 02/04 | G |  | IV | x |  |  |  |  | EPI_ISL_82452 |
| 180 | 30/03 | 01/04 | G |  | I |  |  | x | x | x | EPI_ISL_82453 |
| 182 | 01/04 | 02/04 | G |  |  |  |  |  | x | x | EPI_ISL_82454 |
| 183 | 31/03 | 03/04 | G |  | II |  |  |  |  |  | EPI_ISL_68296 |
| 184 | 31/03 | 01/04 | G |  | IV | x |  |  |  |  | EPI_ISL_82455 |
| 185 | 02/04 | 04/04 | G |  | IV | x |  |  |  |  | EPI_ISL_82456 |
| 186 | 02/04 | 04/04 | C |  | III | x | x | x | x | x | EPI_ISL_68341 |
| 188^+^ | 29/03 | 30/03 | G |  | II |  |  |  |  |  | EPI_ISL_82457 |
| 190 | 03/04 | 04/04 | G |  |  |  |  |  | x | x | EPI_ISL_68324 |
| 191* | 03/04 | 05/04 | L | d1 |  |  |  |  | x | x | EPI_ISL_68275 |
| 192* | 03/04 | 05/04 | L |  |  |  |  |  | x | x | EPI_ISL_82458 |
| 193* | 04/04 | 06/04 | L |  |  |  |  |  | x | x | EPI_ISL_82459 |
| 194^+^ | 05/04 | 05/04 | G |  | II |  |  |  |  |  | EPI_ISL_68297 |
| 195 | 06/04 | 07/04 | G |  | IV | x |  |  |  |  | EPI_ISL_68342 |
| 196* | 06/04 | 06/04 | L |  |  |  |  |  | x | x | EPI_ISL_68325 |
| 197* | 07/04 | 08/04 | L |  |  |  |  |  | x | x | EPI_ISL_82460 |
| 199 | 07/04 | 08/04 | G |  | II |  |  |  |  |  | EPI_ISL_68298 |
| 201 | 09/04 | 11/04 | C |  | III | x | x | x | x | x | EPI_ISL_68345 |
| 202* | 07/04 | 10/04 | L |  |  |  |  |  | x | x | EPI_ISL_68348 |
| 203 | 10/04 | 12/04 | L | d3 |  |  |  |  | x | x | EPI_ISL_68327 |
| 204* | 10/04 | 11/04 | L | d4 |  |  |  |  | x | x | EPI_ISL_68326 |
| 205 | 09/04 | 11/04 | L | d5 |  |  |  |  | x | x | EPI_ISL_68316 |
| 206 | 09/04 | 11/04 | L | d6 |  |  |  |  | x | x | EPI_ISL_82461 |
| 207 | 11/04 | 13/04 | L | d2 |  |  |  |  | x | x | EPI_ISL_68318 |
| 208^+^ | 10/04 | 10/04 | G |  | II |  |  |  |  |  | EPI_ISL_68350 |
| 211 | 13/04 | 14/04 | L |  |  |  |  |  | x | x | EPI_ISL_82462 |
| 213 | 12/04 | 14/04 | L |  |  |  |  |  | x | x | EPI_ISL_68288 |
| **Table S1** Description of the H7N7 virus isolates used in this study (continued). | | | | | | | | | | | |
| Farm code | Sampling date | culling date | Location | Deletion | Cluster | HA A143T | NA N308S | NA A346V | NA T442A | NA P458S | GISAID accession |
| 214 | 12/04 | 15/04 | L | d7 |  |  |  |  | x | x | EPI_ISL_68328 |
| 216 | 14/04 | 15/04 | L |  |  |  |  |  | x | x | EPI_ISL_68349 |
| 217 | 14/04 | 18/04 | L |  |  |  |  |  | x | x | EPI_ISL_68276 |
| 218 | 15/04 | 18/04 | L |  | III | x | x | x | x | x | EPI_ISL_68343 |
| 219* | 16/04 | 17/04 | L | d3 |  |  |  |  | x | x | EPI_ISL_68320 |
| 220 | 16/04 | 18/04 | C |  | III | x | x | x | x | x | EPI_ISL_82463 |
| 221 | 18/04 | 18/04 | L | d7 |  |  |  |  | x | x | EPI_ISL_82464 |
| 222* | 18/04 | 19/04 | L |  | III | x | x | x | x | x | EPI_ISL_68344 |
| 223 | 18/04 | 21/04 | L |  |  |  |  |  | x | x | EPI_ISL_68277 |
| 224 | 05/04 | 21/04 | L | d3 |  |  |  |  | x | x | EPI_ISL_68315 |
| 225 | 20/04 | 22/04 | L |  |  |  |  |  | x | x | EPI_ISL_68286 |
| 226 | 20/04 | 22/04 | L |  |  |  |  |  | x | x | EPI_ISL_82465 |
| 227 | 22/04 | 24/04 | L | d3 |  |  |  |  | x | x | EPI_ISL_68329 |
| 228 | 21/04 | 23/04 | L | d3 |  |  |  |  | x | x | EPI_ISL_68321 |
| 229 | 21/04 | 23/04 | L |  |  |  |  |  | x | x | EPI_ISL_82466 |
| 230 | 22/04 | 24/04 | L |  |  |  |  |  | x | x | EPI_ISL_82467 |
| 231 | 18/04 | 23/04 | L |  |  |  |  |  | x | x | EPI_ISL_68278 |
| 232 | 23/04 | 25/04 | L |  |  |  |  |  | x | x | EPI_ISL_68322 |
| 233 | 24/04 | 26/04 | C |  | III | x | x | x | x | x | EPI_ISL_68346 |
| 234 | 24/04 | 26/04 | L | d3 |  |  |  |  | x | x | EPI_ISL_68351 |
| 235 | 25/04 | 28/04 | C |  | III | x | x | x | x | x | EPI_ISL_82468 |
| 236 | 25/04 | 29/04 | C |  | III | x | x | x | x | x | EPI_ISL_82469 |
| 238^+^ | 05/05 | 05/05 | S |  | III | x | x | x | x | x | EPI_ISL_68347 |
| 239^+^ | 07/05 | 11/05 | L |  |  |  |  |  | x | x | EPI_ISL_68287 |
| 240 | 25/03 | 27/03 | L |  | I |  |  | x | x | x | EPI_ISL_82470 |
|  |  |  |  |  |  |  |  |  |  |  |  |
|  |  |  |  |  |  |  |  |  |  |  |  |

Farm codes correspond to codes given to farms infected during the H7N7 outbreak (F1-F241), following the sequence of report of infection. Dates (day/month/year) correspond to date virus samples were taken from the infected farm, and the date birds were culled. Location corresponds to the 4 infected areas delimited in the main text and Figure 1. Deletions: d1 (nucleotide position 169 to 220 in NA), d2 (169-231), d3 (169-237), d4 (161-238), d5 (166-240), d6 (149-223), d7 (172-237). Clusters I-IV are transmission clusters indentified from the phylogenetic analyses. Presence of five amino acid changes associated with increased pathogenicity in mammalian hosts is indicated for each farm isolate. G, Gelderland; L, Limburg; C, Central area; S, Southwest area. (*) indicates turkey farms; (^+^) indicates back-yard poultry farms; all the other farms are chicken farms.
