# Supplementary material for: Evolutionary Analysis of Inter-Farm Transmission Dynamics in a Highly Pathogenic Avian Influenza Epidemic
Source: PLoS Pathog. 2011 Jun 23;7(6):e1002094. doi: 10.1371/journal.ppat.1002094 (PMC3121798; doi:10.1371/journal.ppat.1002094)
Supplement: Table S2 — Summary statistics of the BMCMC analyses. (DOC) [file ppat.1002094.s005.doc]

**Table S2** Summary statistics of the BMCMC analyses.

|  | **Clock** | **Demographic model** | | | |  |
| --- | --- | --- | --- | --- | --- | --- |
| **Gene** | **model** | **Constant size** | **Exponential** | **Expansion** | **Skyline** | **Lognormal** |
|  |  | **Marginal LnL** | | | |  |
| HA | CLOCK | -3229.55 | -3230.35 | -3234.73 | -3230.88 | -3230.89 |
|  | UCED | -3218.47 | **-3212.10** | -3222.45 | -3217.06 | -3220.19 |
|  | UCLD | -3232.63 | -3229.19 | -3232.20 | -3228.98 | -3229.23 |
| NA | CLOCK | -2634.76 | -2634.44 | -2636.53 | -2633.66 | -2639.22 |
|  | UCED | **-2620.57** | **-2620.19** | **-2620.87** | **-2618.51** | **-2619.04** |
|  | UCLD | -2631.09 | -2629.31 | -2630.71 | -2625.37 | -2628.89 |
| PB2 | CLOCK | -4079.1 | -4081.64 | -4082.51 | -4079.68 | -4080.54 |
|  | UCED | **-4060.39** | **-4058.79** | **-4057.71** | **-4058.52** | **-4058.54** |
|  | UCLD | -4068.94 | -4072.15 | -4070.02 | -4067.67 | -4068.85 |
|  |  | **Mean substitution rate (x10^-2^)** | | | |  |
| HA | CLOCK | 1.36 | 1.19 | 1.31 | 1.41 | 1.15 |
|  | UCED | 1.14 | **1.18** | 1.3 | 1.47 | 1.12 |
|  | UCLD | 1.26 | 1.19 | 1.28 | 1.45 | 1.12 |
| NA | CLOCK | 0.99 | 0.96 | 0.96 | 1.13 | 1.03 |
|  | UCED | **0.94** | **1.02** | **0.94** | **1.14** | **0.98** |
|  | UCLD | 0.98 | 0.99 | 0.97 | 1.08 | 1.01 |
| PB2 | CLOCK | 0.53 | 0.54 | 0.52 | 0.5 | 0.52 |
|  | UCED | **0.5** | **0.54** | **0.51** | **0.52** | **0.48** |
|  | UCLD | 0.51 | 0.55 | 0.52 | 0.49 | 0.5 |
|  |  | **Mean TMRCA** | | | |  |
| HA | CLOCK | 24/01/03 | 26/01/03 | 12/01/03 | 21/02/03 | 09/02/03 |
|  | UCED | 05/01/03 | **15/01/03** | 09/01/03 | 21/02/03 | 30/01/02 |
|  | UCLD | 16/01/03 | 24/01/03 | 21/01/03 | 19/02/03 | 05/02/03 |
| NA | CLOCK | 23/12/02 | 31/12/02 | 27/11/02 | 28/01/03 | 28/12/02 |
|  | UCED | **4/12/02** | **25/12/02** | **27/11/02** | **24/01/03** | **12/12/02** |
|  | UCLD | 10/12/02 | 24/12/02 | 11/12/02 | 25/01/03 | 20/12/02 |
| PB2 | CLOCK | 13/11/02 | 02/11/02 | 25/09/02 | 01/12/02 | 12/10/02 |
|  | UCED | **02/09/02** | **20/10/02** | **01/09/02** | **04/11/02** | **04/09/02** |
|  | UCLD | 24/10/02 | 08/11/02 | 10/10/02 | 10/11/02 | 14/09/02 |

CLOCK, strict molecular clock model; UCED, uncorrelated exponential distributed relaxed clock model; UCLD, uncorrelated lognormal distributed relaxed clock model; Marginal LnL, sampling estimator of the marginal likelihood according to Suchard *et al.* (2003). In bold are the best-fit models and parameter values chosen using the natural log Bayes factor, which is the difference of natural log marginal log likelihoods between models (lnBF > 2.99 is considered significantly different). For both NA and PB2, there was no significantly better demographic model, so we chose the exponential growth model in order to use the same model as for the HA dataset. Substitution rates in number of substitution/site/year. TMRCA, Time since most recent ancestor. Dates are presented in day/month/year.

*References:* Suchard MA, Kitchen CM, Sinsheimer JS, Weiss RE (2003) Hierarchical phylogenetic models for analyzing multipartite sequence data. Syst Biol 52: 649-664.
