# Supplementary material for: Evolutionary Analysis of Inter-Farm Transmission Dynamics in a Highly Pathogenic Avian Influenza Epidemic
Source: PLoS Pathog. 2011 Jun 23;7(6):e1002094. doi: 10.1371/journal.ppat.1002094 (PMC3121798; doi:10.1371/journal.ppat.1002094)
Supplement: Table S3 — List of farm isolates sharing identical HA, NA and/or PB2 sequences. Group names refer to names given in phylogenetic trees in Figure S2A–C. (DOC) [file ppat.1002094.s006.doc]

**Table S3** List of farm isolates sharing identical HA, NA and/or PB2 sequences. Group names refer to names given in phylogenetic trees in Figure S2A-C.

| **Group Name** | **Isolate names** |
| --- | --- |
| **HA** |  |
| Group1 | F18, F33, F36, F62, F68, F167, F176, F192, F197, F203, F205, F206, F207, F211, F219, F224, F228, F232, F237, F242 |
| Group2 | F23, F24, F28, F56, F74, F80 |
| Group3 | F39, F63, F69, F70, F84, F92, F110, F123, F129, F138, F157 |
| Group4 | F16, F19, F52, F65 |
| Group5 | F122, F161, F164, F171, F182, F188 |
| Group6 | F38, F54, F64, F105, F111, F113, F117, F132, F134, F135, F143, F146, F160, F162, F165, F166, F170, F183, F194, F199 |
| Group7 | F32, F49, F58, F71, F131 |
| Group8 | F201, F218, F222, F236 |
| Group9 | F45, F96, F156, F185 |
| Group10 | F1, F2, F5, F8, F10, F12, F14, F15, F17, F20, F21, F25, F29, F31, F37, F41, F42, F43, F50, F55, F60, F67, F76, F91, F99, F116, F121, F130, F191, F193, F217, F223, F231 |
| **NA** |  |
| Group1 | F38, F54, F64, F111, F113, F117, F122, F132, F146, F154, F155, F161, F162, F164, F165, F168, F169, F171, F174, F182, F188, F194, F199 |
| Group2 | F134, F135, F160, F160, F166 |
| Group3 | F94, F96, F144, F184, F195 |
| Group4 | F2, F5, F10, F21, F22, F23, F24, F25, F27, F28, F35, F42, F43, F60, F67, F80, F91, F100, F116 |
| Group5 | F39, F63, F69, F70, F92, F110, F129, F138, F140, F148, F157, F240 |
| Group6 | F76, F142, F179, F186, F201, F218, F220, F222, F233, F236, F238 |
| Group7 | F15, F16, F19, F29, F37, F52, F89, F121 |
| Group8 | F8, F17, F20, F30, F36, F46, F50, F51, F57, F61, F68, F78, F82, F95, F103, F107, F125, F145, F167, F175, F202, F213, F216, F217, F223, F229, F231, F239, F242 |
| **PB2** |  |
| Group1 | F1, F12, F21, F22, F31, F41, F43, F45, F48, F60, F91, F94, F96, F100, F141, F144, F152, F156, F177, F184, F195 |
| Group2 | F23, F24, F27, F32 |
| Group3 | F76, F99, F130, F178 |
| Group4 | F38, F54, F64, F90, F105, F113, F126, F134, F155, F159, F160, F162, F166, F168, F169, F170, F194, F199 |
| Group5 | F122, F154, F161, F164, F171, F182, F208 |
| Group6 | F58, F71, F87, F142, F179, F186, F201,F218, F220, F233, F235, F236, F238 |
| Group7 | F46, F51, F61, F65, F66 |
| Group8 | F15, F16, F19, F29, F37, F39, F52, F69, F70, F84, F92, F102, F108, F129, F140, F180, F240 |
| Group9 | F78, F125, F175, F202 |
| Group10 | F18, F33, F36, F62, F68, F103, F118, F167, F190, F191, F205, F207, F214, F217, F221, F223, F231 |
| Group11 | F197, F211, F232, F242 |
