# Supplementary material for: Evolutionary Analysis of Inter-Farm Transmission Dynamics in a Highly Pathogenic Avian Influenza Epidemic
Source: PLoS Pathog. 2011 Jun 23;7(6):e1002094. doi: 10.1371/journal.ppat.1002094 (PMC3121798; doi:10.1371/journal.ppat.1002094)
Supplement: Table S4 — List of primers and protocols used for PCR amplification and sequencing of HA, NA and PB2 genes. (DOC) [file ppat.1002094.s007.doc]

**Table S4** List of primers and protocols used for PCR amplification and sequencing of HA, NA and PB2 genes.

| **PCR amplification** | |
| --- | --- |
| **Gene** | **Primer sequences (5’-3’)** |
| HA | HA2F*- ATAAGAATGCGGCCGCTATTGCTCTTCAGCCAGCAAAAGCAGGGGATAC |
|  | HA3R- AATCTTAAGCGGCCGCATATGCTCTTCGATTAGTAGAAACAAGGGTGTTTTTCCAAAC |
| NA | SN7F*- TATTGCTCTTCAGCCAGCAAAAGCAGGGTGATYGAGAATG |
|  | SHANSN6N7R- ATATGCTCTTCGATTAGTAGAAACAAGGGTKTTTT |
| PB2 | SPB2F- TATTGCTCTTCAGCCAGCAAAAGCAGGTG |
|  | SPB2R- ATATGCTCTTCGATTAGTAGAAACAAGGTCGTTT |
| **Sequencing reactions** | |
| Gene | Primer sequences (5’-3’) |
|  |  |
| HA | HA18 For- CACCTACAGCGGAATAAGAAC  HA19For- GATTGCTATCATAGTGGAGG  HA21Rev- GTTTGACAGGAGCCATTTCATC  HA23Rev*- CCATCTTCTTCGGCATTCTC |
| NA | NA28For- GGAACGATTCATGACAGAAC  NA29For- CACAUUGGAGCACACAAGUAG  NA31Rev- AGTGCCCAATGGAGTGGAGATG  NA33Rev*- GAAACCCGAATCCCTTCAC |
| PB2 | PB2-38For- CGCAGGAUGUAAUCAUGG  PB2-39For- UUUCAAAAGGACAAGUGGGUC  PB2-40For- CCUGAAGAGGUUAGUGAAACAC  PB2-41Rev- GUGCUGGGGACAUUUGACACUG  PB2-42Rev- TAGTTGAACACAGGGGAGTTGC  PB2-44Rev- CTTCTCTTTTGACAGATGACCC  PB2-45Rev- TTCATTTGGGAAAACGACCTCC |

PCR amplification of the three genes was performed in a 50μl volume using the PCR Expand high fidelity kit (Roche Diagnostics Indianapolis IN, USA) with the concentration of reagents advised by the manufacturer and 0.2µM of each primer. The PCR program used was 94°C for 1 min, followed by 40 cycles of 94°C for 10 sec, 55°C for 15 sec and 72°C for 80 sec, and finally 72°C for 2 min. The primers used for PCR amplification were also used for the sequencing reaction in addition to the primers listed above. (*) Primers used for PCR amplification and sequencing reaction in the cloning analysis.
